# Associations of combined accelerated biological aging and genetic susceptibility with incidence of heart failure in a population‐based cohort study

**DOI:** 10.1111/acel.14430

**Published:** 2024-12-11

**Authors:** Hao Zhao, Xuening Zhang, Yanzhi Li, Wanxin Wang, Wenjian Lai, Wenjing Zhang, Kai Kang, Xiali Zhong, Lan Guo

**Affiliations:** ^1^ Department of Medical Statistics and Epidemiology, School of Public Health Sun Yat‐Sen University Guangzhou China; ^2^ Guangdong Provincial Key Laboratory of Food, Nutrition and Health Sun Yat‐Sen University Guangzhou China; ^3^ Department of Epidemiology and Health Statistics, School of Public Health, Cheeloo College of Medicine Shandong University Jinan China; ^4^ Cardiovascular Department, the First Affiliated Hospital Fujian Medical University Fuzhou China; ^5^ Guangdong Provincial Key Laboratory of Food, Nutrition and Health, Department of Toxicology, School of Public Health Sun Yat‐Sen University Guangzhou China

**Keywords:** biological age, genetic susceptibility, health behavior, heart failure, public health

## Abstract

The global aging population raises concerns about heart failure (HF), yet its association with accelerated biological age (BA) remains inadequately understood. We aimed to examine the longitudinal association between BA acceleration and incident HF risk, assess its modifying effect on genetic susceptibility, and how much BA acceleration mediates the impact of modifiable health behaviors on incident HF. We analyzed 274,608 UK Biobank participants without HF at baseline. Two BA accelerations (Biological Age Acceleration [BioAgeAccel] and Phenotypic Age Acceleration [PhenoAgeAccel]) were calculated by regressing clinical biomarker‐based BA on chronological age, with higher values indicating accelerated aging. Health behavior scores were computed based on diet, physical activity, tobacco/nicotine, sleep, and BMI. Genetic risk scores (GRS) were calculated by 12 HF‐associated loci. During a median follow‐up of 13.5 years, 8915 HF cases were documented. Each standard deviation increase in BioAgeAccel and PhenoAgeAccel was associated with an increased incident HF risk, yielding HRs of 1.45 (95% CI, 1.42–1.48) and 1.42 (95% CI, 1.40–1.45), respectively. Participants with high GRS and highest quartile of BioAgeAccel had an HR of 2.69 (95% CI, 2.42–2.99), and for PhenoAgeAccel, an HR of 2.83 (95% CI, 2.52–3.18), compared to those with low GRS, and lowest quartile. Additive interactions were observed between GRS and BA accelerations. Health behaviors reduced HF risk, with 21.1% (95% CI, 19.5%–22.8%) mediated by decreased BioAgeAccel and 20.9% (95% CI, 19.5%–22.6%) by decreased PhenoAgeAccel. Accelerated BA is associated with an increased incident HF risk, with an additive effect when combined with genetic susceptibility. Maintaining health behaviors may help mitigate BA aging and reduce HF risk.

## Graphical abstract

Our study demonstrates that higher levels of Biological Age Acceleration (BioAgeAccel) and Phenotypic Age Acceleration (PhenoAgeAccel), both derived from routine clinical and blood biomarkers, are significantly associated with an increased risk of incident heart failure (HF). Furthermore, combining these aging metrics with genetic risk scores improves the ability to identify individuals at higher risk of incident HF. Adopting five healthy behaviors, such as eating better, being more active, quitting tobacco, getting healthy sleep, and managing weight, may help mitigate biological aging acceleration and reduce incident HF risk.

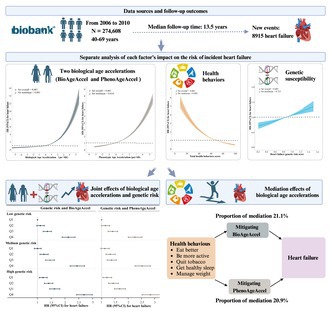

**Abbreviations**
AHA: American Heart Association
AP: Attributable proportion due to interaction
BA: biological age
BioAgeAccel: Biological Age Acceleration
BMI: body mass index
CIs: confidence intervals
CRP: C‐reactive protein
FEV1: forced expiratory volume in 1 s
GRS: genetic risk score
GWAS: genome‐wide association studies
HF: heart failure
HRs: hazard ratios
IMD: Indices of Multiple Deprivation
NHANES: National Health and Nutrition Examination Surveys
PAF: population attributable fraction
PhenoAgeAccel: Phenotypic Age Acceleration
RCS: restricted cubic splines
RERI: relative excess risk due to interaction
SBP: systolic blood pressure
SD: standard deviations

## 1 INTRODUCTION

Heart Failure (HF) is a multi‐faceted and life‐threatening syndrome characterized by significant morbidity and mortality, reduced functional capacity, diminished quality of life, and substantial healthcare costs (Savarese et al., 2023). With the ongoing global aging population and increased life expectancy, the issue of HF has become an increasingly severe public health challenge (Roger, 2021; Siddiqi et al., 2022). Common risk factors, such as ischemic heart disease, atrial fibrillation, hypertension, and diabetes, are closely associated with advancing chronological age (Savarese et al., 2023). However, while chronological age is widely recognized as a major risk factor for HF, it does not fully account for the physiological differences among individuals, nor does it explain the significant variations in HF risk among individuals of the same age.

Biological age (BA) is a concept with greater physiological relevance, encompassing information from multiple biomarkers, offering a more accurate representation of an individual's physiological status and risk of age‐related diseases and death (Jylhävä et al., 2017). Recently, various BA measures, including telomere length, deficit‐accumulation frailty indices, epigenetic clocks based on DNA methylation markers, and algorithms combining information on multiple clinical biomarkers, have been proposed and validated (Diebel & Rockwood, 2021; Jylhävä et al., 2017; van der Harst et al., 2007), with algorithms integrating standard clinical parameters proving to be among the most accurate predictors of incidence and mortality rates, and are relatively easy to obtain and widely applicable (Belsky et al., 2018; Li et al., 2020). Previous studies have found a variety of single biomarkers from blood chemistries (e.g., albumin (Zhuang et al., 2020), C‐reactive protein [CRP] (Burger et al., 2023), glucose (Norris et al., 2022)), and other clinical data (e.g., systolic blood pressure [SBP] (Tsao et al., 2023), forced expiratory volume [FEV1] (Eckhardt et al., 2022)) have been associated with incident HF risks. To date, two distinct methods for measuring BA based on clinical biomarkers: Biological Age (BioAge), which is calculated using the Klemera‐Doubal method to represent the average biological state for a given chronological age within a reference population (Klemera & Doubal, 2006), and Phenotypic Age (PhenoAge), which estimates BA based on the average biological state associated with specific mortality risk levels in a reference population (Liu et al., 2018). The corresponding BA accelerations, Biological Age Acceleration (BioAgeAccel), and Phenotypic Age Acceleration (PhenoAgeAccel), are determined by regressing their respective BA measures against chronological age, with higher values indicating accelerated aging. Both algorithms have been validated in multi‐ethnic cohorts of older adults. However, there remains a lack of research examining the dose–response relationship between these BA accelerations, derived from routinely collected clinical biomarkers, and incident HF risk.

Genetic risk scores (GRS) derived from genome‐wide association studies (GWAS) have been established as effective tools for quantifying an individual's genetic risk for HF (Shah et al., 2020). Although genetic susceptibility to HF is typically considered an inherent factor, the potential influence of accelerated BA on the association between genetic susceptibility on HF risk warrants further investigation. Exploring the combined effects and interactions between BA accelerations and GRS may yield valuable insights into identifying high‐risk populations for HF and refining predictive and preventive strategies. Additionally, while chronological aging is inevitable, mitigating BA acceleration is crucial for improving health outcomes (Galkin et al., 2023). The American Heart Association (AHA) has recently updated its cardiovascular health metrics, which encompasses five modifiable health behaviors: eating better, being more active, quitting tobacco, getting healthy sleep, and managing weight (Ma et al., 2023). However, the extent to which adherence to these health behaviors reduces the risk of incident HF and the mediating roles of the two BA accelerations in this relationship remain unclear.

This study aimed to utilize data from the UK Biobank prospective cohort to construct two measures of BA acceleration: BioAgeAccel and PhenoAgeAccel. We evaluated their dose–response relationships with the risk of incident HF. Additionally, we explored the combined effects of BA accelerations and GRS on the risk of incident HF. Finally, we assessed the extent to which the two BA accelerations mediated the impact of modifiable health behaviors on the risk of incident HF.

## 2 MATERIALS AND METHODS

### 2.1 Study design and participants

The UK Biobank is a prospective population‐based cohort study, which recruited over 500,000 participants aged 40–69 at 22 distinct assessment centers across Scotland, England, and Wales from 2006 to 2010 (Fry et al., 2017). Participants supplied a diverse set of health‐related information through touchscreen surveys, physical measurements, and the submission of biological specimens, including blood, urine, and saliva samples for biochemical analysis and genotyping. Additional details regarding the UK Biobank protocol can be accessed online at www.ukbiobank.ac.uk. The UK Biobank has approval from the North West Multi‐centre Research Ethics Committee. All participants provided informed written consent.

**FIGURE 1:**
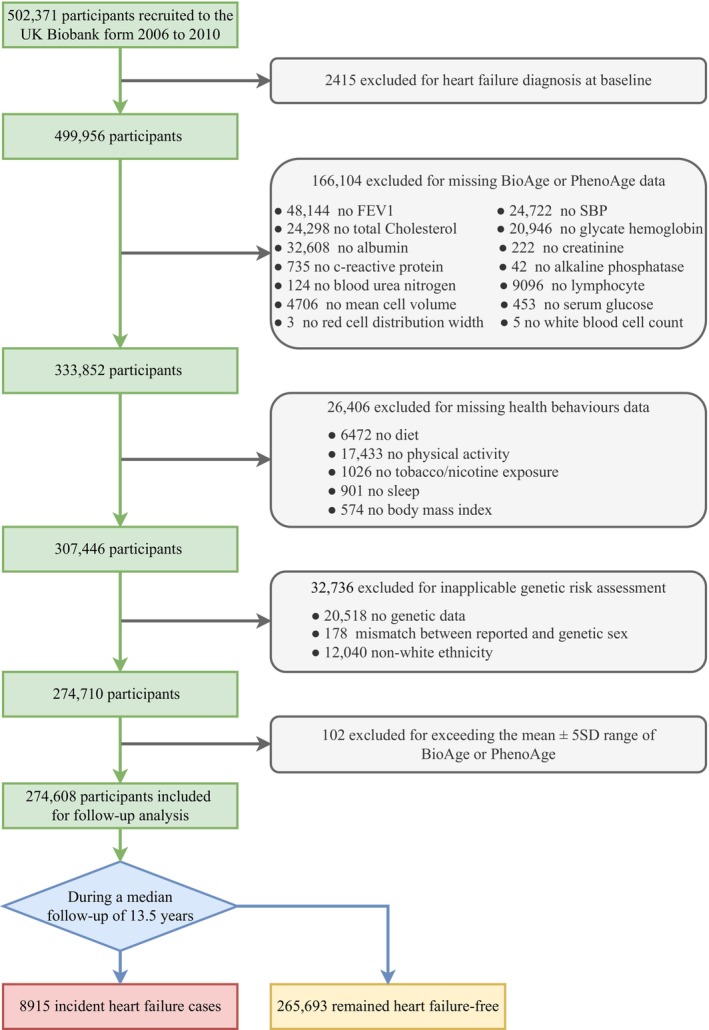
Flow diagram for inclusion of participants in this study. BioAge, Biological Age; PhenoAge, Phenotypic Age; FEV1, forced expiratory volume in 1 s; SBP, systolic blood pressure; SD, standard deviations.

Among the 502,371 participants with available data in the current study, we excluded individuals who had a prior history of HF (*N* = 2415) at the baseline assessment. Additionally, participants at the baseline with missing data pertaining to BA (*N* = 166,104), health behavior assessment (*N* = 26,406), and those lacking genetic information, or with discrepancies between reported and genetic sex, or individuals of non‐white ethnicity (*N* = 32,736) were excluded from the study. Furthermore, we identified and excluded additional participants (*N* = 102) whose BA values were considered outliers, defined as values falling outside the range of ±5 standard deviations (SD) from the mean. After applying these exclusion criteria, a total of 274,608 participants remained eligible for inclusion in the primary analysis (Figure 1). This study was conducted in accordance with the Strengthening the Reporting of Observational Studies in Epidemiology (STROBE) guidelines (Vandenbroucke et al., 2007).

### 2.2 Assessment of biological age and biological age acceleration

We employed two of the most rigorously validated BA algorithms compatible with the available data from the UK Biobank: BioAge and PhenoAge algorithms, both based on clinical and blood chemistry metrics (Gao et al., 2023; Kwon & Belsky, 2021). Briefly, BioAge was calculated using the Klemera‐Doubal method and included forced expiratory volume in 1 s, SBP, and seven blood chemistry parameters: albumin, alkaline phosphatase, blood urea nitrogen, creatinine, CRP, glycated hemoglobin, and total cholesterol. PhenoAge included nine blood chemistries, four of which overlapped with BioAge (albumin, alkaline phosphatase, creatinine, and CRP), along with glucose, mean cell volume, red cell distribution width, white blood cell count, and lymphocyte proportion. A full list of the biomarkers and their corresponding UK Biobank data fields is provided in Table S1. Detailed explanations of the calculations and interpretations of these two BA measures have been summarized elsewhere (Kwon & Belsky, 2021; Mak et al., 2023). The computation of BA values was executed using the R package “BioAge” (https://github.com/dayoonkwon/BioAge).

The BioAge algorithm was computed using the Klemera‐Doubal method, which involved a series of regression analyses of biomarkers against chronological age (Klemera & Doubal, 2006). This method aligned an individual's average physiological profile with that of participants in the US National Health and Nutrition Examination Surveys (NHANES) III, which served as the training dataset. Notably, the algorithm parameters were estimated separately for males and females. The BioAge formula is represented as follows:

$$\text{BioAge}=\frac{\sum_{i=1}^{9}\left(x_{i}-q_{i}\right)\frac{k_{i}}{s_{i}^{2}}+\frac{\mathrm{CA}}{s_{\mathrm{BA}}^{2}}}{\sum_{i=1}^{9}{\left(\frac{k_{i}}{s_{i}}\right)}^{2}+\frac{1}{s_{\mathrm{BA}}^{2}}}$$

In this calculation, *x* represents the value of biomarker i for an individual. CA is chronological age. For each biomarker i, the parameters *k*, *q*, and s are estimated from a regression of chronological age on the biomarker in the reference sample, where *k* is the intercept, *q* is the slope, and s is the root mean squared error. $s_{\mathrm{BA}}$ is a scaling factor equal to the square root of the variance in chronological age explained by the biomarker set in the reference sample.

The PhenoAge algorithm was derived from an extensive analysis of mortality risks (Liu et al., 2018). Initially, the original PhenoAge algorithm was developed using an elastic‐net Gompertz regression model of mortality data on 42 biomarkers from the NHANES III dataset. To create a more concise and effective age estimator, the model selected a subset of biomarkers by choosing the lambda that minimized the mean squared error, ultimately identifying nine key clinical biomarkers. The PhenoAge formula is represented as follows:

$$\text{PhenoAge}=141.50225+\frac{\ln\left[-0.0055305\times \ln\left(1-\text{mortality risk}\right)\right]}{0.090165}$$

$$\text{mortality risk}=1-e^{-e^{\mathrm{xb}}\left[\exp\left(120\times \gamma \right)-1\right]/\gamma }$$

γ=0.007692696

$$\begin{array}{c} \mathrm{xb}=-19.90667-0.03359\times \text{albumin}+0.00951\times \text{creatinine}+0.19532\\ \times \text{glucose}+0.09537\times \ln\left(\mathrm{CRP}\right)-0.01200\times \text{lymphocyte percentage}\\ +0.02676\times \text{mean cell volume}+0.33062\times \mathrm{red}\;\text{cell distribution width}\\ +0.00187\times \text{alkaline phosphatase}+0.05542\times \text{white blood cell count}\\ +0.08035\times \text{chronological}\;\mathrm{age} \end{array}$$

Based on the above methods, the procedure for constructing the BA involved training the algorithms in NHANES III and projecting BA measurements onto NHANES IV, followed by application to the UK Biobank dataset (Kwon & Belsky, 2021). To measure deviations in BA from chronological age, we calculated the “age acceleration” for both BioAge and PhenoAge, representing the residuals from regressing BioAge and PhenoAge against chronological age. Elevated values of BioAgeAccel and PhenoAgeAccel represent advanced BA. The two age acceleration values were separately standardized (mean = 0, SD = 1) for comparability in subsequent analyses.

### 2.3 Assessment of health behaviors

The health behavior score was developed based on five behaviors from the AHA Life's Essential 8 components: diet, physical activity, tobacco/nicotine exposure, sleep, and body mass index (BMI) (Lloyd‐Jones et al., 2022). The diet was assessed using 10 dietary components, including fruit, vegetable, whole grains, fish, dairy, vegetable oils, refined grains, processed meats, unprocessed meats, and sugar‐sweetened beverages, with 1 point given for meeting each intake recommendation (Said et al., 2018; Zhang et al., 2021) (Table S2). The diet score was subsequently mapped to a 0–100 scale based on the percentiles of the number of recommendations achieved, as shown in Table S3, with higher scores indicating a healthier eating pattern. Physical activity was assessed by quantifying the total weekly minutes spent in moderate and vigorous activities. Tobacco/nicotine exposure was assessed based on smoking status (never, former, or current) and secondhand smoke exposure (yes or no). Former smokers were further classified by smoking frequency and quit duration. Sleep duration was determined through self‐reported average nightly hours of sleep. BMI was calculated as weight in kilograms divided by height in meters squared. Comprehensive details and the scoring algorithm for each behavior component are provided in Table S3, with each of the five components scored on a 0–100 scale. The overall health behavior score was computed by summing these scores, dividing by 5, and also ranging from 0 to 100 points. Subsequently, participants were categorized into three categories: unfavorable, intermediate, and favorable behaviors, determined by their placement within the tertile distribution of the health behavior score.

### 2.4 Genetic risk score calculation for HF

The UK Biobank conducted comprehensive SNP genotyping, imputation, and quality control procedures. The construction of the GRS for HF relied on 12 independent variants associated with HF at the genome‐wide significance threshold (*p* < 5 × 10^−8^), as identified in a prior GWAS (Shah et al., 2020) (Table S4). The weighted GRS method is expressed as follows:

$$\mathrm{GRS}=\sum_{i=1}^{m}\beta_{i}\times {\mathrm{SNP}}_{i}$$

where $\beta_{i}$ represents the natural logarithm of the odds ratio per allele for HF‐related SNPs, while ${\mathrm{SNP}}_{i}$ is recoded as 0, 1, or 2 based on the number of risk alleles, and m is equal to 12. Participants were categorized into three groups: low genetic risk (tertile 1), intermediate genetic risk (tertile 2), and high genetic risk (tertile 3) for HF.

### 2.5 Measurements of covariates

In our analyses, we incorporated several covariates based on previous research findings. These covariates included age, sex, years of education, income levels, employment status, Indices of Multiple Deprivation (IMD), and alcohol consumption. To estimate years of education, we utilized the International Standard for Classification of Education codes, which were aligned with participants' self‐reported highest qualification in the UK Biobank dataset (Carter et al., 2019). Years of education were categorized as 10 years or fewer, 11–15 years, or more than 15 years. Annual household income levels were divided into four categories: level 1 (<£18,000), level 2 (£18,000–£30,999), level 3 (£31,000–£51,999), and level 4 (>£52,000). Employment status was categorized as employed (including those in paid or self‐employment, doing unpaid or voluntary work, and full or part‐time students) and unemployed. The IMD is a measure derived from a qualitative study conducted by the UK government to assess levels of deprivation across local areas (McLennan et al., 2011). Unlike the Townsend Index, the IMD provides a more comprehensive evaluation by incorporating a broader range of factors. Specifically, the English IMD consists of seven domains: income deprivation; employment deprivation; health deprivation and disability; education, skills and training deprivation; barriers to housing and services; living environment deprivation; crime. These domains are combined into an overall IMD score. For further detail, please refer to the following link: https://biobank.ndph.ox.ac.uk/showcase/label.cgi?id=76. Alcohol consumption was classified into five categories: daily or almost daily, 3–4 times per week, 1–2 times per week, occasionally, and never.

### 2.6 Assessment of outcomes

Baseline prevalent HF cases were identified based on self‐reported information and hospital inpatient records. During follow‐up, HF diagnoses were ascertained using hospital inpatient records (Hospital Episode Statistics for England, Morbidity Records for Scotland, and the Patient Episode Database for Wales) and death register data (National Health Service [NHS] Digital, NHS Central Register, and National Records of Scotland). Specifically, International Classification of Diseases 10th revision (ICD‐10) codes I11.0, I13.0, I13.2, I50.0, I50.1, I50.9, and ICD‐9 code 428 were used to identify participants with HF. At the time of our analyses, the censoring dates for the Hospital Episode Statistics for England, Scottish Morbidity Records for Scotland, and Patient Episode Database for Wales were as follows: 31 October 2022, 31 July 2021, and 28 February 2018, respectively. The follow‐up period was calculated from baseline to the time of HF diagnosis, death, loss to follow‐up, or censoring, whichever occurred first.

### 2.7 Statistical analysis

Baseline summary statistics are presented with means and standard deviations (SD) for continuous variables and proportions for categorical variables. We employed Cox proportional hazards regression models to estimate hazard ratios (HRs) along with their corresponding 95% confidence intervals (CIs) to assess the association between BioAgeAccel or PhenoAgeAccel and incident HF risk. The proportional hazards assumptions were verified by computing scaled Schoenfeld residuals and scrutinizing time‐based log HR plots. Our modeling process included two progressively adjusted models: Model 1 adjusted for age and sex, while Model 2 additionally accounted for the assessment center, years of education, income levels, employment status, IMD, alcohol consumption, and health behavior score. Additionally, we calculated population attributable fraction (PAF), which measures the proportion of disease cases attributed to a specific exposure in the population (Miettinen, 1974). To further explore the dose–response associations between BioAgeAccel or PhenoAgeAccel (as continuous variables) and incident HF risk, we employed restricted cubic splines (RCS) models with three knots (Desquilbet & Mariotti, 2010). Furthermore, we performed a post hoc exploratory subgroup analysis to assess potential variations in different subgroups defined by age, sex, years of education, income levels, alcohol employment status, IMD, and alcohol consumption.

We also assessed the joint associations between BioAgeAccel or PhenoAgeAccel and genetic categories with incident HF risk, while further adjusting for the first 10 genetic principal components and genotype batch. The reference category comprised individuals in the lowest quartile of BioAgeAccel or PhenoAgeAccel and the lowest tertile of genetic susceptibility. Additionally, we employed the relative excess risk due to interaction (RERI) and the attributable proportion due to interaction (AP) to evaluate the additive interaction between genetic risk and the two measures of BA acceleration on the risk of HF.

Mediation analysis was conducted to evaluate whether reductions in BioAgeAccel or PhenoAgeAccel mediated the association between modifiable health behavior score and incident HF risk based on the method proposed by Baron & Kenny, (1986). The mediation effect was considered if the following conditions were satisfied: (1) health behavior score was significantly associated with BioAgeAccel or PhenoAgeAccel; (2) health behavior score was significantly associated with HF risk; (3) BioAgeAccel or PhenoAgeAccel was significantly associated with HF risk; and (4) the association between health behavior score and HF risk was attenuated when BioAgeAccel or PhenoAgeAccel were included in the model. Once these conditions were simultaneously met, the direct and indirect effects were estimated using the “mediation” package in R with 1000 bootstrapped iterations (Tingley et al., 2014). The mediation proportion was also calculated to quantify the extent to which BioAgeAccel or PhenoAgeAccel mediated the relationship between health behavior score and HF risk.

We conducted several sensitivity analyses to account for potential biases. First, we excluded HF cases occurring within 2 years prior to follow‐up to mitigate reverse causality. Second, we further excluded patients with cardiovascular diseases such as coronary heart disease, myocardial infarction, and arrhythmia at baseline to ensure that HF events were more attributable to BA acceleration than other known cardiovascular diseases. Third, given the relatively high proportion of individuals with incomplete BA measurements excluded from the primary analysis, we used median imputation to fill in missing values for individuals with only one missing biomarker out of the nine measured biomarkers in each algorithm. This approach allowed us to assess the relationship between imputed BA acceleration and HF risk. Fourth, we performed stratified analyses by age and follow‐up time using a time‐varying model with interaction terms between BioAgeAccel or PhenoAgeAccel and age (in 5‐year intervals) or between BioAgeAccel or PhenoAgeAccel and follow‐up time (in 3‐year intervals) to calculate HRs for different age and time periods. Fifth, we further adjusted telomere length to examine the collinearity and independence of BioAgeAccel or PhenoAgeAccel and telomere length. Finally, to address potential bias from competing risks such as deaths from other causes, we employed Fine and Gray subdistribution hazards regression models.

All analyses were carried out using R version 4.2.3. A two‐sided *p*‐value of 0.05 or less was considered to indicate statistical significance.

## 3 RESULTS

### 3.1 Baseline characteristics of participants

The study comprised 274,608 participants, with a mean (SD) age at baseline of 56.5 (8.0) years. Among them, 148,120 (53.9%) were female. During a median follow‐up of 13.5 years (3,564,477 person‐years), 8915 new HF events were observed. Table 1 displays the baseline characteristics of the participants stratified by incident HF. Compared with those who did not develop HF, participants who did were generally older in both chronological and BA, predominantly male and unemployed, had lower educational attainment, lower income, higher IMD scores, were either non‐drinkers or consumed alcohol daily, had poorer health behavior scores, and exhibited higher GRS.

**TABLE 1:** Baseline characteristics of study participants by incident heart failure status.

Characteristic	Total participants (*n* = 274,608)	Incident HF (*n* = 8915)	Non‐HF (*n* = 265,693)	*p*‐value
Age at baseline, years	56.49 (8.04)	62.10 (6.14)	56.30 (8.03)	<0.001
Sex				
Female	148,120 (53.9)	3195 (35.8)	144,925 (54.5)	<0.001
Male	126,488 (46.1)	5720 (64.2)	120,768 (45.5)	
Assessment centre				
England	250,762 (91.3)	8568 (96.1)	242,194 (91.2)	<0.001
Scotland	11,461 (4.2)	214 (2.4)	11,247 (4.2)	
Wales	12,385 (4.5)	133 (1.5)	12,252 (4.6)	
Education, years				
< =10	88,658 (32.3)	4016 (45.0)	84,642 (31.9)	<0.001
11–14	49,357 (18.0)	1513 (17.0)	47,844 (18.0)	
>15	134,831 (49.1)	3293 (36.9)	131,538 (49.5)	
Unknown	1762 (0.6)	93 (1.0)	1669 (0.6)	
Household income, £				
Less than 18,000	49,368 (18.0)	2870 (32.2)	46,498 (17.5)	<0.001
18,000–30,999	60,727 (22.1)	2217 (24.9)	58,510 (22.0)	
31,000–51,999	64,842 (23.6)	1448 (16.2)	63,394 (23.9)	
Greater than 52,000	66,314 (24.1)	976 (10.9)	65,338 (24.6)	
Unknown	33,357 (12.1)	1404 (15.7)	31,953 (12.0)	
Employment status				
Employed	169,544 (61.7)	3242 (36.4)	166,302 (62.6)	<0.001
Unemployed	104,432 (38.0)	5652 (63.4)	98,780 (37.2)	
Unknown	632 (0.2)	21 (0.2)	611 (0.2)	
Indices of multiple deprivation, quartile				
Q1 (Least deprived)	72,409 (26.4)	1961 (22.0)	70,448 (26.5)	<0.001
Q2	70,143 (25.5)	2036 (22.8)	68,107 (25.6)	
Q3	66,099 (24.1)	2204 (24.7)	63,895 (24.0)	
Q4 (Most deprived)	58,995 (21.5)	2505 (28.1)	56,490 (21.3)	
Unknown	6962 (2.5)	209 (2.3)	6753 (2.5)	
Alcohol intake				
Never	16,936 (6.2)	851 (9.5)	16,085 (6.1)	<0.001
Occasionally	58,433 (21.3)	2071 (23.2)	56,362 (21.2)	
1–2 times a week	72,252 (26.3)	2112 (23.7)	70,140 (26.4)	
3–4 times a week	67,323 (24.5)	1809 (20.3)	65,514 (24.7)	
Daily or almost daily	59,561 (21.7)	2066 (23.2)	57,495 (21.6)	
Unknown	103 (0.0)	6 (0.1)	97 (0.0)	
Total behaviors score	67.05 (13.75)	61.58 (15.04)	67.23 (13.67)	<0.001
Components of behaviors				
Diet score	32.10 (19.17)	31.82 (19.88)	32.11 (19.15)	0.151
Physical activity score	71.54 (38.19)	66.82 (40.74)	71.70 (38.09)	<0.001
Tobacco/nicotine exposure score	71.96 (30.21)	65.90 (32.66)	72.16 (30.11)	<0.001
Sleep health score	89.83 (18.09)	86.80 (20.83)	89.93 (17.98)	<0.001
Body mass index score	69.81 (28.17)	56.56 (30.94)	70.25 (27.97)	<0.001
GRS	0.87 (0.20)	0.89 (0.20)	0.87 (0.20)	<0.001
Biological ages				
BioAge	54.11 (9.16)	61.40 (7.68)	53.87 (9.10)	<0.001
BioAgeAccel	0.00 (4.39)	1.68 (5.12)	−0.06 (4.35)	<0.001
PhenoAge	49.79 (9.41)	58.21 (8.57)	49.51 (9.30)	<0.001
PhenoAgeAccel	0.00 (4.57)	2.68 (5.96)	−0.09 (4.49)	<0.001
Components of biological ages				
FEV1 (L)	2.89 (0.79)	2.63 (0.79)	2.90 (0.78)	<0.001
SBP (mmHg)	137.70 (18.47)	144.31 (19.62)	137.48 (18.39)	<0.001
Total Cholesterol (mg/dL)	221.50 (43.69)	206.51 (47.89)	222.00 (43.45)	<0.001
Glycated hemoglobin (%)	5.42 (0.56)	5.73 (0.90)	5.41 (0.54)	<0.001
Albumin (g/dL)	4.53 (0.26)	4.46 (0.27)	4.53 (0.26)	<0.001
Creatinine (mg/dL)	0.82 (0.17)	0.88 (0.25)	0.81 (0.17)	<0.001
CRP (mg/dL)	0.24 (0.40)	0.37 (0.57)	0.24 (0.40)	<0.001
Alkaline phosphatase (U/L)	82.74 (25.55)	88.37 (29.42)	82.56 (25.39)	<0.001
Blood urea nitrogen (mg/dL)	15.15 (3.72)	16.70 (5.24)	15.10 (3.65)	<0.001
Lymphocyte (%)	28.81 (7.26)	26.77 (7.55)	28.88 (7.24)	<0.001
Mean cell volume (fL)	82.80 (5.22)	83.25 (5.62)	82.79 (5.21)	<0.001
Serum glucose (mmol/L)	5.10 (1.15)	5.51 (1.90)	5.08 (1.11)	<0.001
Red cell distribution width (%)	13.45 (0.92)	13.72 (1.13)	13.44 (0.91)	<0.001
White blood cell count (1000 cells/μL)	6.84 (1.82)	7.36 (2.00)	6.82 (1.81)	<0.001

*Note*: Mean values (standard deviation) for continuous variables and *n* (%) for categorical variables. *p*‐values are derived using either Student's *t* test or Chi‐square test.

Abbreviations: BioAgeAccel, Biological Age Acceleration; CRP, C‐reactive protein; FEV1, forced expiratory volume in 1 s; GRS, genetic risk score; HF, heart failure; PhenoAgeAccel, Phenotypic Age Acceleration; SBP, systolic blood pressure.

In the NHANES IV validation cohort for constructing BA metrics, chronological age was strongly correlated with BioAge (*r* = 0.96) and PhenoAge (*r* = 0.96), while a moderate correlation was observed between BioAgeAccel and PhenoAgeAccel (*r* = 0.45) (Figure S1A). Both BioAgeAccel and PhenoAgeAccel were significantly associated with all‐cause mortality, further supporting the reliability of our BA metrics (Table S5). Similarly, in the UK Biobank, chronological age was strongly correlated with BioAge (*r* = 0.88) and PhenoAge (*r* = 0.87), while a weak correlation was found between BioAgeAccel and PhenoAgeAccel (*r* = 0.30) (Figure S1B).

### 3.2 Associations between biological age accelerations and the risk of incident HF

Table 2 presents the associations between BA accelerations and risk of incident HF. After adjusting for age, sex, assessment center, years of education, income levels, employment status, IMD, alcohol consumption, and health behavior score, participants in the highest quartile (Q4) of BioAgeAccel had a 128% higher risk of incident HF compared to those in the lowest quartile (Q1) (HR: 2.28, 95% CI: 2.15–2.43), with 22.05% of incident HF cases attributed to it in the study population. Similarly, those in the highest quartile (Q4) of PhenoAgeAccel showed a 131% increased HF risk compared to the lowest quartile (Q1) (HR: 2.31, 2.16–2.47), with 25.65% of incident HF cases attributed to it in the study population. Each SD increase in BioAgeAccel was associated with a 45% higher risk of incident HF (HR: 1.45, 95% CI: 1.42–1.48), while each SD increase in PhenoAgeAccel was associated with a 42% higher risk of incident HF (HR: 1.42, 95% CI: 1.40–1.45).

**TABLE 2:** Association between biological age measures and the risk of incident heart failure in UK Biobank.

Characteristic	*N*	Cases/person‐years	Model 1		Model 2		PAF (%)
			HR (95% CI)	*p*‐value	HR (95% CI)	*p*‐value	
BioAgeAccel							
Q1	68,652	1580/900055	1 (Reference)		1 (Reference)		
Q2	68,652	1744/897386	1.29 (1.20–1.38)	<0.001	1.17 (1.09–1.25)	<0.001	2.84
Q3	68,652	2090/892502	1.66 (1.55–1.77)	<0.001	1.39 (1.30–1.48)	<0.001	6.58
Q4	68,652	3501/874534	3.04 (2.86–3.22)	<0.001	2.28 (2.15–2.43)	<0.001	22.05
Per SD increase	274,608	8915/3564477	1.60 (1.57–1.63)	<0.001	1.45 (1.42–1.48)	<0.001	
PhenoAgeAccel							
Q1	68,652	1216/908826	1 (Reference)		1 (Reference)		
Q2	68,652	1616/900894	1.26 (1.17–1.36)	<0.001	1.18 (1.09–1.27)	<0.001	2.77
Q3	68,652	2051/892853	1.54 (1.44–1.66)	<0.001	1.34 (1.25–1.45)	<0.001	5.84
Q4	68,652	4032/861904	3.05 (2.86–3.26)	<0.001	2.31 (2.16–2.47)	<0.001	25.65
Per SD increase	274,608	8915/3564477	1.54 (1.51–1.56)	<0.001	1.42 (1.40–1.45)	<0.001	

*Note*: Model 1: Adjusted for age and sex. Model 2: Further adjusted for assessment center, years of education, income levels, employment status, IMD, alcohol consumption, and health behavior score.

Abbreviations: BioAgeAccel, Biological Age acceleration; CI, confidence interval; HR, hazard ratio; PAF, population attributable fraction; PhenoAgeAccel, Phenotypic Age acceleration; SD, standard deviations.

**FIGURE 2:**
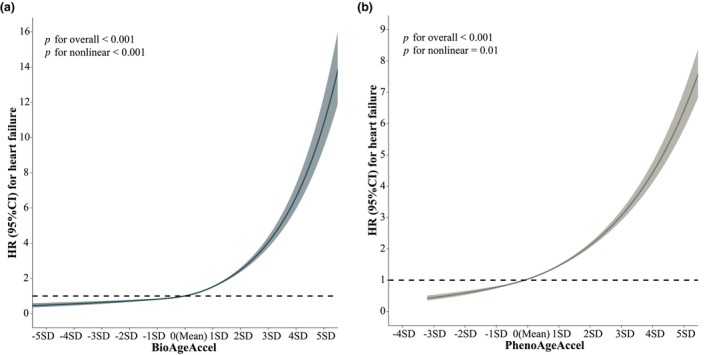
Association between biological age accelerations and the risk of incident heart failure using restricted cubic splines (RCS) models with three knots. (a) BioAgeAccel on the risk of incident heart failure; (b) PhenoAgeAccel on the risk of incident heart failure. The models were adjusted for age, sex, assessment center, years of education, income levels, employment status, Index of Multiple Deprivation, alcohol consumption, and health behavior score. Solid lines represent HRs, while shaded areas represent 95% CIs. BioAgeAccel, Biological Age Acceleration; CI, confidence interval; HR, hazard ratio; PhenoAgeAccel, Phenotypic Age Acceleration; SD, standard deviations.

Utilizing RCS analyses, we detected monotonic and non‐linear exposure‐response associations between BioAgeAccel and PhenoAgeAccel and the risk of incident HF (all *p* for overall association <0.05, *P* for non‐linear association <0.05; Figure 2). Specifically, the risk increases significantly when BioAgeAccel and PhenoAgeAccel are greater than zero, while values less than zero are associated with a protective effect. Subgroup analyses indicated that both BioAgeAccel and PhenoAgeAccel heightened the risk of incident HF across various subgroups (Figure S2).

In the sensitivity analysis, after excluding participants diagnosed with incident HF within the first 2 years of follow‐up, excluding participants diagnosed with any cardiovascular disease at baseline, using median imputation for BA calculations with nine biomarkers where only one value was missing, and applying competing risks models, no significant changes were observed in the associations between both BioAgeAccel and PhenoAgeAccel and the risk of incident HF (Table S6–S9). Furthermore, although the proportional hazards assumption was considered reasonable, as evidenced by the approximately parallel log(HR) curves for BioAgeAccel and PhenoAgeAccel over follow‐up time (Figure S3), we conducted stratified time‐varying models and found that the association between BioAgeAccel and PhenoAgeAccel and the risk of incident HF remained consistent across different age groups (in 5‐year intervals) and follow‐up intervals (in 3‐year intervals), aligning with the main analysis results without any abnormal variations (Figure S4). Additionally, we found no significant collinearity between BA accelerations and telomere length, and the significant associations between BA accelerations and HF risk remained consistent after adjusting for telomere length (Table S10).

### 3.3 Joint relations and interactions of biological age accelerations and genetic susceptibility with the risk of incident HF

In separate analyses of genetic risk, participants with high GRS had a 23% increased risk of incident HF compared to those with low GRS (HR 1.23, 95% CI 1.17–1.30). We observed a linear positive association between GRS and the risk of incident HF (*p* for overall association <0.05, *P* for non‐linear association >0.05; Figure S5 and Table S11). For each 1‐point increase in GRS, the risk of HF increased by 62% (HR 1.62, 95% CI 1.47–1.80).

**FIGURE 3:**
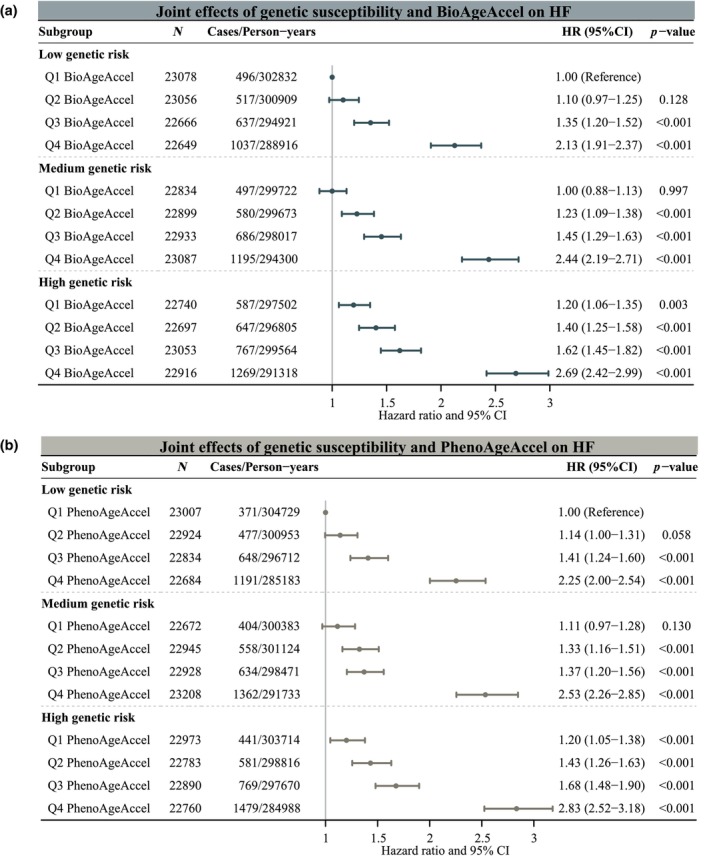
Joint effects of biological age accelerations and genetic risk on the risk of incident heart failure. (a) BioAgeAccel and genetic risk on the risk of incident heart failure; (b) PhenoAgeAccel and genetic risk on the risk of incident heart failure. The models were adjusted for age, sex, assessment center, years of education, income levels, employment status, Index of Multiple Deprivation, alcohol consumption, health behavior score, genotyping array, and the first 10 genetic principal components. BioAgeAccel, Biological Age Acceleration; CI, confidence interval; HR, hazard ratio; PhenoAgeAccel, Phenotypic Age Acceleration.

In the joint analysis, participants with high GRS and Q4 BioAgeAccel had the highest HF risk, with an HR of 2.69 (95% CI, 2.42–2.99), compared to those with low GRS and Q1 BioAgeAccel. Similarly, participants with high GRS and Q4 PhenoAgeAccel had an HR of 2.83 (95% CI, 2.52–3.18) compared to those with low GRS and Q1 PhenoAgeAccel (Figure 3). Although no significant multiplicative interaction between GRS and BA accelerations on HF risk was observed, a significant additive interaction was detected (Table S12). Specifically, for participants with high GRS and Q4 BioAgeAccel, the RERI was 0.36 (95% CI, 0.13–0.59), indicating that the relative excess risk due to additive interaction was 0.36, accounting for 0.14 (95% CI, 0.05–0.22) of the HF risk in participants with exposure to both high GRS and Q4 PhenoAgeAccel. Similarly, for participants with high GRS and Q4 PhenoAgeAccel, the RERI was 0.37 (95% CI, 0.14–0.60) and the AP was 0.13 (95% CI, 0.05–0.21).

### 3.4 Mediation effects of biological age accelerations

**FIGURE 4:**
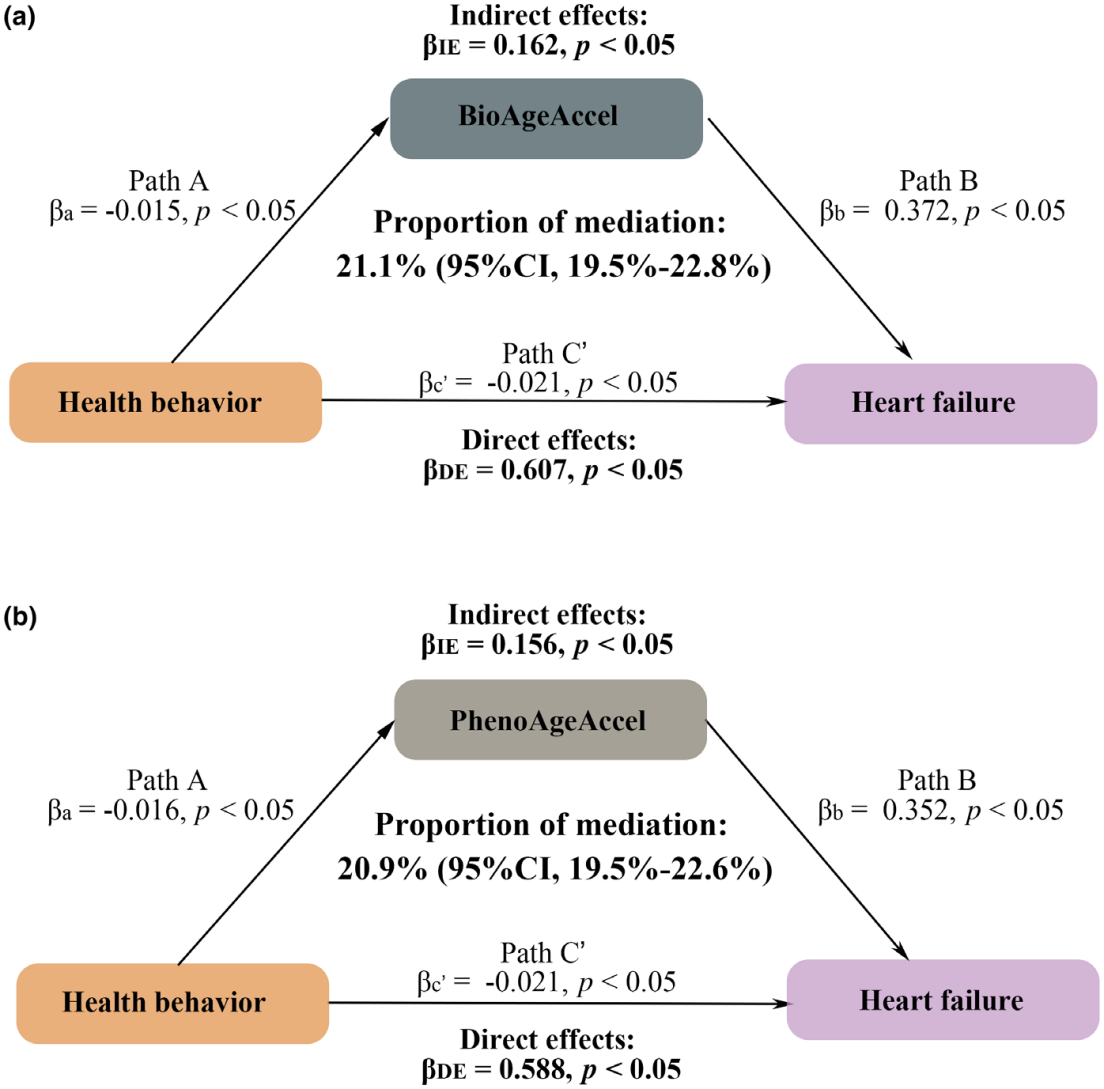
Mediation effect of biological age accelerations on the associations between behaviors score and heart failure. (a) The mediating proportion of BioAgeAccel; (b) The mediating proportion of PhenoAgeAccel. *p*‐values for path A were computed by multiple linear regressions. *p*‐values for path B or path C′ were computed by Cox proportional hazard regressions. *p*‐values for IE and DE were computed by the casual mediation analysis. Covariates included age, sex, assessment center, years of education, income levels, employment status, Index of Multiple Deprivation, and alcohol consumption. 95% confidence intervals were generated from 1000 bootstrap samples. BioAgeAccel, Biological Age Acceleration; CI, confidence interval; DE, direct effect; IE, indirect effect; PhenoAgeAccel, Phenotypic Age Acceleration.

We observed a linear negative association between the health behavior score, based on five health behaviors, and the risk of incident HF (*p* for overall association <0.05, *p* for non‐linear association >0.05; Figure S6). Specifically, each 10‐point increase in the health behavior score was associated with a 24% reduction in HF risk (HR 0.76, 95% CI 0.75–0.78) (Table S13). Additionally, Path A showed that health behavior score was negatively associated with BioAgeAccel and PhenoAgeAccel (all *p* < 0.05). Furthermore, Path B showed that BioAgeAccel and PhenoAgeAccel had harmful effects on incident HF risk (all *p* < 0.05). Through mediation analysis, we found that BioAgeAccel mediated 21.1% (95% CI, 19.5%–22.8%) of the association between behavior scores and incident HF risk, while PhenoAgeAccel mediated 20.9% (95% CI, 19.5%–22.6%) of this relationship (Figure 4).

## 4 DISCUSSION

Given the escalating prevalence of HF driven by the global aging trend, our study sought to prospectively assess the association between two types of BA acceleration and the risk of incident HF, while also exploring its modifying role on genetic susceptibility and the extent to which BA accelerations mediate the impact of modifiable health behaviors on HF risk. Analyzing data from 274,608 UK Biobank participants, we observed significant associations between higher BioAgeAccel or PhenoAgeAccel and an increased risk of incident HF. Notably, we found an additive interaction between BA accelerations and genetic risk, with individuals with the high GRS and Q4 BA acceleration having the highest HF risk compared to those with the low GRS and Q1 BA acceleration. Furthermore, our findings suggested that adherence to healthy behaviors may reduce the risk of HF, partially mediated by mitigating BA accelerations.

Prior studies have primarily utilized telomere length and DNA methylation as measures of BA, with initial findings suggesting that HF patients exhibited significantly shorter telomere lengths and that DNA methylation age was associated with HF risk (Bey et al., 2023, 2024; Wong et al., 2008). However, these methods rely heavily on omics data, which poses challenges for standardization and broader clinical application. In our study, we constructed two distinct BA metrics based on routine clinical biomarkers. Both metrics share common markers, such as CRP, albumin, alkaline phosphatase, and creatinine, which reflect systemic inflammation, liver function, and kidney function. Notably, BioAge is associated with clinical biomarkers indicative of cardiovascular and metabolic health, including lung function (FEV1), blood pressure (SBP), lipid metabolism (total cholesterol), and glucose metabolism (HbA1c). In contrast, PhenoAge focuses more on inflammation and immune function. Despite capturing different dimensions of aging (Kuo et al., 2021), both metrics were found to significantly increase incident HF risk. Each standard deviation increase in BioAgeAccel and PhenoAgeAccel was associated with a 45% and 42% higher risk of HF, respectively. This consistency underscores the robust predictive value of clinical biomarker‐based BA accelerations in assessing HF risk. Moreover, these BA metrics were initially developed using data from the NHANES‐III cohort (Kwon & Belsky, 2021), which is more ethnically diverse than the UK Biobank cohort used in this study. These findings suggest that monitoring BA acceleration not only aids in the early identification of individuals at high risk for HF, enabling timely clinical interventions, but also highlights its potential as a valuable tool for HF prevention and surveillance in public health.

The potential biological mechanisms linking BA indicators derived from routine clinical and biomarker data to the risk of HF involve multiple systems. First, both BA indicators reflect liver and kidney function. As critical organs for metabolism and detoxification, impaired liver and kidney function can lead to the accumulation of toxins, electrolyte imbalances, and metabolic dysfunction, all of which place additional strain on the heart and increase the risk of HF (House et al., 2019; Xanthopoulos et al., 2019). Second, BioAge primarily captures cardiovascular metabolic disturbances. Reduced lung function may decrease oxygen exchange efficiency, exacerbating cardiac load (Eckhardt et al., 2022). Hypertension directly increases cardiac pressure, elevating HF risk (Pfeffer, 2017). Dysregulated lipid metabolism can lead to atherosclerosis, further raising cardiovascular disease risk, while impaired glucose metabolism contributes to HF through complications such as diabetes (Low Wang et al., 2016). Lastly, PhenoAge focuses on assessing inflammation and immune status. Chronic inflammation can damage cardiomyocytes and promote fibrosis, altering the heart's structure and function (Adamo et al., 2020). Concurrently, abnormal immune activation may further damage cardiac tissues and contribute to functional decline (Zhang et al., 2017). This excessive immune response, combined with persistent systemic inflammation, not only impairs cardiac health but also elevates the overall risk of HF through widespread inflammatory processes.

Accelerated BA not only serves as an independent predictor of HF but can also be combined with genetic risk to identify high‐risk individuals who are most likely to experience adverse outcomes without intervention. We observed a significant additive interaction between genetic risk and BA acceleration, which further amplifies the risk of HF. Since genetic risk is an inherent and largely unmodifiable factor, mitigating accelerated BA is crucial to reducing the HF risk associated with high genetic predisposition. Previous research, such as a study using data from the Health and Retirement Study, has indicated that 29.2% of the BA variance can be explained by 11 domains, with behavioral factors accounting for the largest proportion (9.2%) (Liu et al., 2019). Our findings further emphasize the critical role of health behaviors in reducing BA accelerations and HF risk. Adhering to the latest AHA recommendations (Lloyd‐Jones et al., 2022), such as eating better, being more active, quitting tobacco, getting healthy sleep, and managing weight, may slow BA acceleration through mechanisms like preserving cardiovascular health, reducing chronic inflammation, and supporting metabolic function. Given the inevitability of chronological aging, reducing BA acceleration through health behavior interventions is critical, especially in the context of global aging. This strategy may be beneficial for promoting healthy aging and decreasing HF incidence.

Our study presents several notable strengths. Firstly, we leveraged a large, prospective cohort from the UK Biobank and employed two published and validated clinical‐based BA algorithms, BioAge, and PhenoAge. This dual approach enabled us to explore the impact of BA on HF from different aging perspectives, thereby enhancing the robustness of our findings. Additionally, our investigation delved into the intricate relationships between accelerated BA, health behaviors, and genetic susceptibility, providing a comprehensive theoretical foundation for HF prevention and intervention strategies. However, there are also notable limitations to our study. First, our analysis relied solely on baseline clinical biomarkers. Although we utilized time‐varying models in the sensitivity analyses, we were unable to assess how changes in BA over time might influence HF risk. Second, the UK Biobank data does not differentiate between specific HF subtypes, such as HF with reduced ejection fraction and HF with preserved ejection fraction. Future research should aim to examine how accelerated BA affects these HF subtypes to refine risk assessments. Third, the UK Biobank dataset includes a significant number of participants with missing clinical data necessary for BA calculation. To reduce selection bias, we conducted a sensitivity analysis using imputed biomarkers to estimate BA acceleration, with results consistent with the primary analysis. Additionally, stratified analyses across different demographic subgroups also demonstrated that both BioAgeAccel and PhenoAgeAccel increased HF risk within each subgroup. Fourth, as with other observational studies, while we have meticulously adjusted for major risk factors, we cannot entirely rule out the possibility of residual or unmeasured confounding. Lastly, the study population predominantly consists of individuals of White ethnicity from the UK Biobank, which may limit the generalizability of our findings to other populations. Future research should aim to address these limitations and explore these relationships in a broader and more diverse context.

Our study reveals that higher levels of BioAgeAccel or PhenoAgeAccel are significantly associated with an increased risk of HF and can be used in combination with GRS for risk stratification of HF. Elevated BA acceleration may also amplify the impact of genetic factors on HF risk. Given the robustness and accessibility of BA metrics derived from conventional clinical and blood biomarkers, these indicators hold promise as novel clinical composite biomarkers for guiding precise prevention and management in high‐risk populations. Furthermore, adhering to the five recommended healthy lifestyle behaviors by the latest AHA, including eating better, being more active, quitting tobacco, getting healthy sleep, and managing weight, may help mitigate BA acceleration and reduce the risk of HF.

## AUTHOR CONTRIBUTIONS

H.Z., X. Zhang, Y. L., X. Zhong, and L.G. conceived the study and developed the statistical analysis plan. H.Z., X. Zhang, and Y. L., conducted the statistical analysis and wrote prepared the first draft of the manuscript. W.W., K.K., W.L., W.Z., X. Zhong, and L.G. contributed to the critical revision of the manuscript for important intellectual content. All authors reviewed and approved the final manuscript.

## FUNDING INFORMATION

This work was supported by the Natural Science Foundation of Guangdong Province (Grant Nos. 2021A1515111184, 2023A1515012255, 2022A1515012333), and Natural Science Foundation of China (Grant No. 82473652).

## CONFLICT OF INTEREST STATEMENT

The authors declare no conflict of interest.

## Supporting information


**Data S1:** Supporting Information.

## Data Availability

Data are available in a public, open access repository. This research has been conducted using the UK Biobank Resource under Application Number 95114. The UK Biobank data are available on application to the UK Biobank (www.ukbio bank.ac.uk/).
