# Supplementary material for: Associations of combined accelerated biological aging and genetic susceptibility with incidence of heart failure in a population‐based cohort study
Source: Aging Cell. 2024 Dec 11;24(4):e14430. doi: 10.1111/acel.14430 (PMC11984684; doi:10.1111/acel.14430)
Supplement: Supplementary file 1 — Data S1: Supporting Information. [file ACEL-24-e14430-s001.docx]

## Supplementary Materials

**Associations of combined accelerated biological aging and genetic susceptibility with incidence of heart failure in a population-based cohort study**

## Table of Content

[**Table S1. Biomarkers included in the biological age measures and their field IDs in UK Biobank** 2](#_Toc177973410)

[**Table S2. Components of Dietary Recommendations for Adherence to the American Heart Association Guidelines in the UK Biobank** 3](#_Toc177973411)

[**Table S3. Methods for evaluating each individual healthy behavior in the UK Biobank.** 5](#_Toc177973412)

[**Table S4. Information of genetic variants associated with heart failure in the UK Biobank.** 7](#_Toc177973413)

[**Table S5. Association between biological age measures and the risk of all-cause death in NHANES4** 8](#_Toc177973414)

[**Table S6. Association between biological age accelerations and the risk of incident heart failure after excluding participants diagnosed with heart failure within the first two years of follow-up.** 9](#_Toc177973415)

[**Table S7. Association between biological age accelerations and the risk of incident heart failure after excluding participants diagnosed with any cardiovascular disease at baseline.** 10](#_Toc177973416)

[**Table S8. Association between biological age accelerations and the risk of incident heart failure for nine biomarkers with only one missing value imputed using the median.** 11](#_Toc177973417)

[**Table S9. Association between biological age accelerations and the risk of incident heart failure** **using competitive risk model** 12](#_Toc177973418)

[**Table S10. Association between biological age accelerations and the risk of incident heart failure** **after further adjusting telomere length** 13](#_Toc177973419)

[**Table S11. Association between genetic risk and the incident risk of heart failure** 14](#_Toc177973420)

[**Table S12. Additive interactions between biological age accelerations and genetic risk score and the risk of heart failure** 15](#_Toc177973421)

[**Table S13. Association between health behaviors and the risk of incident heart failure** 16](#_Toc177973422)

[**Figure S1. Correlations between biological age measures and chronological age in UK Biobank.** 17](#_Toc177973423)

[**Figure S2. Association of biological age accelerations and the risk of incident heart failure stratified by potential risk factors.** 18](#_Toc177973424)

[**Figure S3. Scaling Schoenfeld residuals and time-based logarithmic HR plots of biological age accelerations.** 19](#_Toc177973425)

[**Figure S4. Association between biological age accelerations and the risk of incident heart failure using time-varying model with interaction terms between BioAgeAccel or PhenoAgeAccel and age (in 5-year intervals) (A and C) or between BioAgeAccel or PhenoAgeAccel and follow-up time (in 3-year intervals) (B and D).** 20](#_Toc177973426)

[**Figure S5. Association between genetic risk scores and the risk of incident heart failure using restricted cubic splines models with three knots.** 21](#_Toc177973427)

[**Figure S6. Association between total health behavior scores and the risk of incident heart failure using restricted cubic splines models with three knots.** 22](#_Toc177973428)

**Table S1. Biomarkers included in the biological age measures and their field IDs in UK Biobank**

| **Variables** | **Field ID** | **Included in BioAge** | **Included in PhenoAge** |
| --- | --- | --- | --- |
| FEV1 (L) | 3063 | Yes |  |
| SBP (mm Hg) | 4080 | Yes |  |
| Total Cholesterol (mg/dL) | 30690 | Yes |  |
| Glycated hemoglobin (%) | 30750 | Yes |  |
| Blood urea nitrogen (mg/dL) | 30670 | Yes |  |
| Albumin (g/dL) | 30600 | Yes | Yes |
| Creatinine (mg/dL) | 30700 | Yes | Yes |
| C-reactive protein (mg/dL) | 30710 | Yes | Yes |
| Alkaline phosphatase (U/L) | 30610 | Yes | Yes |
| Lymphocyte (%) | 30180 |  | Yes |
| Mean cell volume (fL) | 30270 |  | Yes |
| Serum glucose (mmol/L) | 30740 |  | Yes |
| Red cell distribution width (%) | 30070 |  | Yes |
| White blood cell count (1000 cells/uL) | 30000 |  | Yes |

BioAge, Biological Age; PhenoAge, Phenotypic Age; FEV1, forced expiratory volume in one second; SBP, systolic blood pressure.

**Table S2. Components of Dietary Recommendations for Adherence to the American Heart Association Guidelines in the UK Biobank**

| **Diet component** | **Field IDs** | **Amount per serving** | **Intake goal** |
| --- | --- | --- | --- |
| Fruit | 1309 (pieces fresh fruit/day)  1319 (pieces dried fruit/day) | 1309 – 1 piece  1319 – 5 pieces | 3 servings/day |
| Vegetable | 1289 (tablespoons cooked vegetables/day)  1299 (salad/raw vegetables/day) | 3 heaped tablespoons | 3 servings/day |
| Whole grains | 1438, 1448 (whole meal/wholegrain bread slices/week)  1458, 1468 (bran/oat/muesli cereal bowls/week) | 1438/1448 – 1 slice/day  1458/1468 – 1 bowl/day | 3 servings/day |
| (Shell)fish | 1329 (oily fish/week)  1339 (non-oily fish/week) | Once/week | ≥2 servings/week |
| Dairy | 1408 (cheese/week)  1418 (milk type) | 1408 – 1 piece/day  1418 – 1 glass/day if consumption of any type of milk | 2 servings/day |
| Vegetable oils | 1428 (Flora Pro-Active/Benecol spread)  2654 (Flora Pro-Active/Benecol, soft margarine -, olive oil based -, polyunsaturated/sunflower oil based -, other low/reduced fat spread)  1438 (bread slices/week) | 1 serving/day if in combination with eating at least 2 slices of bread (ID 1438) | 2 servings/day |
| Refined grains | 1438, 1448 (white, brown, other bread slices/week)  1458, 1468 (biscuit, other cereals/week) | 1438/1448 – 1 slice/day  1458/1468 – 1 bowl/day | ≤2 servings/day |
| Processed meats | 1349 (processed meat/week or daily)  3680 (age when last ate meat) | 1349 – 1 piece/day  3680 – 0 pieces/day if indicated having never eaten meat | ≤1 serving/week |
| Unprocessed meats | 1359 (poultry/week or day)  1369 (beef/week or day)  1379 (lamb or mutton/week or day)  1389 (pork/week or day)  3680 (age when last ate meat) | 1359-1389 – once/week  3680 – 0 pieces/day if indicated having never eaten meat | ≤2 servings/week |
| Sugar-sweetened beverages | 6144 (never consumes drinks containing sugar) | Only 0 servings were possible here. | Don’t drink |

Field IDs and serving sizes used per diet component in UK Biobank with available data from the general baseline questionnaire. If participants achieved the intake goal, they were considered to have an adequate intake of the diet component.

Scoring criteria for dietary recommendations: 1: If intake goal met; 0: If intake goal not met. (Range: 0-10)

**Table S3. Methods for evaluating each individual healthy behavior in the UK Biobank.**

| **healthy behavior metric** | **Quantification of healthy behavior metric** | |
| --- | --- | --- |
|  | **Status** | **Scoring points** |
| Diet | Achieving 7-10 dietary recommendations - 95th percentile (top/ideal diet)  Achieving 5-6 dietary recommendations - 75th–94th percentile  Achieving 3-4 dietary recommendations - 50th–74th percentile  Achieving 2 dietary recommendations - 25th–49th percentile  Achieving 0-1 dietary recommendations - 1st–24th percentile (bottom/ least ideal quartile) | 100  80  50  25  0 |
| Physical activity | ≥150 minutes  120–149 minutes  90–119 minutes  60–89 minutes  30–59 minutes  1–29 minutes  0 minutes | 100  90  80  60  40  20  0 |
| Tobacco/nicotine exposure | Never smoker  Former smoker, quit ≥ 5 years  Former smoker, quit 1–<5 years  Former smoker, quit <1 years  Current smoker | 100  75  50  25  0 |
|  | Subtract 20 points (unless score is 0) for living with active indoor smoker in home. Moreover, because the information on the specific time to quit smoking was only available in participants who indicated “smoked on most or all days in the past”. We  regard the participants who indicated “smoked occasionally in the past” as equivalent to “Former smoker, quit 1–<5 years”; We regard the participants who indicated “just tried once or twice in the past” as equivalent to “Former smoker, quit ≥ 5 years”. | |
| Sleep health | 7–<9 hours  9–<10 hours  6–<7 hours  5–<6 or ≥10 hours  4–<5 hours  <4 hours | 100  90  70  40  20  0 |
| BMI | <25.0 kg/m2  25.0–29.9 kg/m2  30.0–34.9 kg/m2  35.0–39.9 kg/m2  ≥40.0 kg/m2 | 100  70  30  15  0 |

**Table S4. Information of genetic variants associated with heart failure in the UK Biobank.**

| **Chromosome** | **SNP** | **Nearest gene** | **Effect allele** | **Effect size** |
| --- | --- | --- | --- | --- |
| 5 | rs11745324 | KLHL3 | G | 0.048790164 |
| 6 | rs140570886 | LPA | C | 0.21511138 |
| 9 | rs1556516 | 9p21/CDKN2B-AS1 | C | 0.058268908 |
| 4 | rs17042102 | PITX2, FAM241A | A | 0.113328685 |
| 10 | rs17617337 | BAG3 | C | 0.058268908 |
| 6 | rs4135240 | CDKN1A | T | 0.048790164 |
| 10 | rs4746140 | SYNPO2L, AGAP5 | G | 0.067658648 |
| 12 | rs4766578 | ATXN2 | T | 0.039220713 |
| 6 | rs55730499 | LPA | T | 0.104360015 |
| 16 | rs56094641 | FTO | G | 0.048790164 |
| 9 | rs600038 | ABO, SURF1 | C | 0.058268908 |
| 1 | rs660240 | CELSR2 | C | 0.058268908 |

**Table S5. Association between biological age measures and the risk of all-cause death in NHANES4**

| **Characteristic** | **Cases/Person-years** | **Model 1** | |
| --- | --- | --- | --- |
|  |  | **HR (95% CI)** | ***P*-value** |
| **BioAgeAccel** |  |  |  |
| Q1 | 35/169281 | 1 (Reference) |  |
| Q2 | 49/167731 | 1.57 (1.02-2.42) | 0.042 |
| Q3 | 33/167781 | 1.13 (0.7-1.82) | 0.613 |
| Q4 | 73/165242 | 2.15 (1.43-3.21) | <0.001 |
| Per SD increase | 190/670035 | 1.35 (1.19-1.53) | <0.001 |
| **PhenoAgeAccel** |  |  |  |
| Q1 | 497/965844 | 1 (Reference) |  |
| Q2 | 501/887898 | 1.27 (1.12-1.44) | <0.001 |
| Q3 | 543/779021 | 1.65 (1.46-1.86) | <0.001 |
| Q4 | 808/620244 | 2.99 (2.66-3.35) | <0.001 |
| Per SD increase | 2349/3253007 | 1.51 (1.46-1.56) | <0.001 |

BioAgeAccel, Biological Age acceleration; PhenoAgeAccel, Phenotypic Age acceleration; HR, hazard ratio; CI, confidence interval; SD, standard deviations

Model 1: Adjusted for age and sex

**Table S6. Association between biological age accelerations and the risk of incident heart failure after excluding participants diagnosed with heart failure within the first two years of follow-up.**

| **Characteristic** | **N** | **Cases/Person-years** | **Model 1** | | **Model 2** | |
| --- | --- | --- | --- | --- | --- | --- |
|  |  |  | **HR (95% CI)** | ***P*-value** | **HR (95% CI)** | ***P*-value** |
| **BioAgeAccel** |  |  |  |  |  |  |
| Q1 | 68538 | 1499/899518 | 1 (Reference) |  | 1 (Reference) |  |
| Q2 | 68538 | 1655/896934 | 1.29 (1.2-1.38) | <0.001 | 1.17 (1.09-1.25) | <0.001 |
| Q3 | 68537 | 1981/892316 | 1.65 (1.54-1.77) | <0.001 | 1.39 (1.29-1.48) | <0.001 |
| Q4 | 68538 | 3323/875185 | 3.03 (2.85-3.22) | <0.001 | 2.29 (2.15-2.44) | <0.001 |
| Per SD increase | 274151 | 8458/3563953 | 1.6 (1.57-1.63) | <0.001 | 1.45 (1.42-1.48) | <0.001 |
| **PhenoAgeAccel** |  |  |  |  |  |  |
| Q1 | 68538 | 1181/907717 | 1 (Reference) |  | 1 (Reference) |  |
| Q2 | 68538 | 1531/900407 | 1.24 (1.15-1.33) | <0.001 | 1.15 (1.07-1.24) | <0.001 |
| Q3 | 68537 | 1964/892436 | 1.53 (1.42-1.65) | <0.001 | 1.34 (1.24-1.44) | <0.001 |
| Q4 | 68538 | 3782/863393 | 2.97 (2.78-3.17) | <0.001 | 2.25 (2.11-2.41) | <0.001 |
| Per SD increase | 274151 | 8458/3563953 | 1.53 (1.51-1.55) | <0.001 | 1.41 (1.39-1.44) | <0.001 |

BioAgeAccel, Biological Age acceleration; PhenoAgeAccel, Phenotypic Age acceleration; HR, hazard ratio; CI, confidence interval; SD, standard deviations

Model 1: Adjusted for age and sex;

Model 2: Further adjusted for assessment center, years of education, income levels, employment status, Index of Multiple Deprivation, alcohol consumption, and health behavior score

**Table S7. Association between biological age accelerations and the risk of incident heart failure after excluding participants diagnosed with any cardiovascular disease at baseline.**

| **Characteristic** | **N** | **Cases/Person-years** | **Model 1** | | **Model 2** | |
| --- | --- | --- | --- | --- | --- | --- |
|  |  |  | **HR (95% CI)** | ***P*-value** | **HR (95% CI)** | ***P*-value** |
| **BioAgeAccel** |  |  |  |  |  |  |
| Q1 | 64893 | 1128/853743 | 1 (Reference) |  | 1 (Reference) |  |
| Q2 | 64892 | 1309/851199 | 1.34 (1.24-1.45) | <0.001 | 1.23 (1.13-1.33) | <0.001 |
| Q3 | 64892 | 1550/847426 | 1.69 (1.57-1.83) | <0.001 | 1.44 (1.33-1.56) | <0.001 |
| Q4 | 64893 | 2649/832244 | 3.13 (2.92-3.36) | <0.001 | 2.42 (2.25-2.61) | <0.001 |
| Per SD increase | 259570 | 6636/3384611 | 1.6 (1.57-1.64) | <0.001 | 1.47 (1.43-1.5) | <0.001 |
| **PhenoAgeAccel** |  |  |  |  |  |  |
| Q1 | 64893 | 975/860244 | 1 (Reference) |  | 1 (Reference) |  |
| Q2 | 64892 | 1245/853718 | 1.23 (1.13-1.34) | <0.001 | 1.15 (1.06-1.25) | <0.001 |
| Q3 | 64892 | 1583/847408 | 1.53 (1.41-1.66) | <0.001 | 1.34 (1.24-1.46) | <0.001 |
| Q4 | 64893 | 2833/823241 | 2.77 (2.58-2.99) | <0.001 | 2.14 (1.99-2.31) | <0.001 |
| Per SD increase | 259570 | 6636/3384611 | 1.5 (1.47-1.53) | <0.001 | 1.39 (1.36-1.42) | <0.001 |

BioAgeAccel, Biological Age acceleration; PhenoAgeAccel, Phenotypic Age acceleration; HR, hazard ratio; CI, confidence interval; SD, standard deviations

Model 1: Adjusted for age and sex;

Model 2: Further adjusted for assessment center, years of education, income levels, employment status, Index of Multiple Deprivation, alcohol consumption, and health behavior score

**Table S8. Association between biological age accelerations and the risk of incident heart failure for nine biomarkers with only one missing value imputed using the median.**

| **Characteristic** | **N** | **Cases/Person-years** | **Model 1** | | **Model 2** | |
| --- | --- | --- | --- | --- | --- | --- |
|  |  |  | **HR (95% CI)** | ***P*-value** | **HR (95% CI)** | ***P*-value** |
| **BioAgeAccel** |  |  |  |  |  |  |
| Q1 | 99657 | 2433/1303123 | 1 (Reference) |  | 1 (Reference) |  |
| Q2 | 99657 | 2742/1297176 | 1.27 (1.2-1.34) | <0.001 | 1.15 (1.09-1.21) | <0.001 |
| Q3 | 99656 | 3264/1291212 | 1.61 (1.52-1.69) | <0.001 | 1.34 (1.27-1.42) | <0.001 |
| Q4 | 99657 | 5195/1265518 | 2.79 (2.66-2.93) | <0.001 | 2.08 (1.98-2.19) | <0.001 |
| Per SD increase | 398627 | 13634/5157028 | 1.56 (1.53-1.58) | <0.001 | 1.41 (1.38-1.43) | <0.001 |
| **PhenoAgeAccel** |  |  |  |  |  |  |
| Q1 | 99657 | 1839/1317118 | 1 (Reference) |  | 1 (Reference) |  |
| Q2 | 99657 | 2473/1304384 | 1.28 (1.2-1.36) | <0.001 | 1.19 (1.12-1.26) | <0.001 |
| Q3 | 99656 | 3146/1293049 | 1.59 (1.5-1.69) | <0.001 | 1.39 (1.31-1.47) | <0.001 |
| Q4 | 99657 | 6176/1242478 | 3.14 (2.98-3.31) | <0.001 | 2.36 (2.24-2.5) | <0.001 |
| Per SD increase | 398627 | 13634/5157028 | 1.54 (1.52-1.56) | <0.001 | 1.43 (1.41-1.45) | <0.001 |

BioAgeAccel, Biological Age acceleration; PhenoAgeAccel, Phenotypic Age acceleration; HR, hazard ratio; CI, confidence interval; SD, standard deviations

Model 1: Adjusted for age and sex;

Model 2: Further adjusted for assessment center, years of education, income levels, employment status, Index of Multiple Deprivation, alcohol consumption, and health behavior score

**Table S9. Association between biological age accelerations and the risk of incident heart failure** **using competitive risk model**

| **Characteristic** | **N** | **Cases/Person-years** | **Model 1** | | **Model 2** | |
| --- | --- | --- | --- | --- | --- | --- |
|  |  |  | **HR (95% CI)** | ***P*-value** | **HR (95% CI)** | ***P*-value** |
| **BioAgeAccel** |  |  |  |  |  |  |
| Q1 | 68652 | 1580/900055 | 1 (Reference) | | 1 (Reference) | |
| Q2 | 68652 | 1744/897386 | 1.27 (1.19-1.36) | <0.001 | 1.16 (1.08-1.24) | <0.001 |
| Q3 | 68652 | 2090/892502 | 1.62 (1.52-1.73) | <0.001 | 1.37 (1.28-1.46) | <0.001 |
| Q4 | 68652 | 3501/874534 | 2.88 (2.71-3.06) | <0.001 | 2.19 (2.06-2.33) | <0.001 |
| Per SD increase | 274608 | 8915/3564477 | 1.55 (1.52-1.59) | <0.001 | 1.41 (1.38-1.44) | <0.001 |
| **PhenoAgeAccel** |  |  |  |  |  |  |
| Q1 | 68652 | 1216/908826 | 1 (Reference) | | 1 (Reference) | |
| Q2 | 68652 | 1616/900894 | 1.26 (1.17-1.35) | <0.001 | 1.17 (1.09-1.27) | <0.001 |
| Q3 | 68652 | 2051/892853 | 1.52 (1.42-1.64) | <0.001 | 1.34 (1.24-1.44) | <0.001 |
| Q4 | 68652 | 4032/861904 | 2.89 (2.71-3.09) | <0.001 | 2.2 (2.06-2.36) | <0.001 |
| Per SD increase | 274608 | 8915/3564477 | 1.47 (1.45-1.5) | <0.001 | 1.37 (1.34-1.39) | <0.001 |

BioAgeAccel, Biological Age acceleration; PhenoAgeAccel, Phenotypic Age acceleration; HR, hazard ratio; CI, confidence interval; SD, standard deviations

Model 1: Adjusted for age and sex using competitive risk model;

Model 2: Further adjusted for assessment center, years of education, income levels, employment status, Index of Multiple Deprivation, alcohol consumption, and health behavior score using competitive risk model

**Table S10. Association between biological age accelerations and the risk of incident heart failure** **after further adjusting telomere length**

| **Characteristic** | **N** | **Cases/Person-years** | **Model 3** | |
| --- | --- | --- | --- | --- |
|  |  |  | **HR (95% CI)** | ***P*-value** |
| **BioAgeAccel** |  |  |  |  |
| Q1 | 68652 | 1580/900055 | 1 (Reference) |  |
| Q2 | 68652 | 1744/897386 | 1.17 (1.09-1.25) | <0.001 |
| Q3 | 68652 | 2090/892502 | 1.39 (1.3-1.48) | <0.001 |
| Q4 | 68652 | 3501/874534 | 2.28 (2.14-2.43) | <0.001 |
| Per SD increase | 274608 | 8915/3564477 | 1.45 (1.42-1.48) | <0.001 |
| **PhenoAgeAccel** |  |  |  |  |
| Q1 | 68652 | 1216/908826 | 1 (Reference) |  |
| Q2 | 68652 | 1616/900894 | 1.17 (1.09-1.27) | <0.001 |
| Q3 | 68652 | 2051/892853 | 1.34 (1.25-1.44) | <0.001 |
| Q4 | 68652 | 4032/861904 | 2.3 (2.15-2.46) | <0.001 |
| Per SD increase | 274608 | 8915/3564477 | 1.42 (1.4-1.44) | <0.001 |

BioAgeAccel, Biological Age acceleration; PhenoAgeAccel, Phenotypic Age acceleration; HR, hazard ratio; CI, confidence interval; SD, standard deviations

Model 3: Further adjusted for assessment center, years of education, income levels, employment status, Index of Multiple Deprivation, alcohol consumption, health behavior score, and telomere length.

**Table S11. Association between genetic risk and the incident risk of heart failure**

| **Characteristic** | **N** | **Cases/Person-years** | **Model 1** | | **Model 2** | |
| --- | --- | --- | --- | --- | --- | --- |
|  |  |  | **HR (95% CI)** | ***P*-value** | **HR (95% CI)** | ***P*-value** |
| **Genetic risk** |  |  |  |  |  |  |
| Low genetic risk | 91449 | 2687/1187577 | 1 (Reference) |  | 1 (Reference) |  |
| Medium genetic risk | 91753 | 2958/1191711 | 1.1 (1.04-1.16) | <0.001 | 1.1 (1.04-1.16) | <0.001 |
| High genetic risk | 91406 | 3270/1185189 | 1.23 (1.17-1.3) | <0.001 | 1.23 (1.17-1.3) | <0.001 |
| Per 1 score increase | 274608 | 8915/3564477 | 1.62 (1.46-1.8) | <0.001 | 1.62 (1.47-1.8) | <0.001 |

HR, hazard ratio; CI, confidence interval

Model 1: Adjusted for age and sex;

Model 2: Further adjusted for assessment center, years of education, income levels, employment status, Index of Multiple Deprivation, alcohol consumption, health behavior score, genotyping array, and the first ten genetic principal components

**Table S12. Additive interactions between biological age accelerations and genetic risk score and the risk of heart failure**

| **Characteristic** | **Medium genetic risk** | | **High genetic risk** | |
| --- | --- | --- | --- | --- |
|  | **RERI (95% CI)** | **AP (95% CI)** | **RERI (95% CI)** | **AP (95% CI)** |
| **BioAgeAccel** |  |  |  |  |
| Q1 |  |  |  |  |
| Q2 | 0.13 (-0.05-0.31) | 0.11 (-0.04-0.25) | 0.11 (-0.08-0.31) | 0.08 (-0.05-0.21) |
| Q3 | 0.1 (-0.09-0.3) | 0.07 (-0.06-0.2) | 0.08 (-0.12-0.28) | 0.05 (-0.07-0.17) |
| Q4 | 0.3 (0.09-0.52) | 0.13 (0.04-0.22) | 0.36 (0.13-0.59) | 0.14 (0.05-0.22) |
| **PhenoAgeAccel** |  |  |  |  |
| Q1 |  |  |  |  |
| Q2 | 0.07 (-0.14-0.28) | 0.05 (-0.11-0.21) | 0.09 (-0.13-0.3) | 0.06 (-0.09-0.21) |
| Q3 | -0.15 (-0.37-0.07) | -0.11 (-0.27-0.05) | 0.07 (-0.15-0.29) | 0.04 (-0.09-0.17) |
| Q4 | 0.16 (-0.07-0.39) | 0.06 (-0.03-0.16) | 0.37 (0.14-0.6) | 0.13 (0.05-0.21) |

BioAgeAccel, Biological Age acceleration; PhenoAgeAccel, Phenotypic Age acceleration; RERI, relative excess risk due to interaction; AP, Attributable proportion due to interaction; CI, confidence interval

Adjusted for age, sex, assessment center, years of education, income levels, employment status, Index of Multiple Deprivation, alcohol consumption, behavior scores, genotype batch, and the first ten genetic principal components. To estimate RERI and AP, the lowest of biological age acceleration group and the lowest PRS group were set as reference.

**Table S13. Association between health behaviors and the risk of incident heart failure**

| **Characteristic** | **N** | **Cases/Person-years** | **Model 1** | | **Model 2** | |
| --- | --- | --- | --- | --- | --- | --- |
|  |  |  | **HR (95% CI)** | ***P*-value** | **HR (95% CI)** | ***P*-value** |
| **Health behaviors** |  |  |  |  |  |  |
| Favorable behaviors | 93268 | 4359/1193473 | 1 (Reference) |  | 1 (Reference) |  |
| Intermediate behaviors | 93264 | 2780/1212989 | 0.58 (0.55-0.61) | <0.001 | 0.62 (0.59-0.65) | <0.001 |
| Unfavorable behaviors | 88076 | 1776/1158015 | 0.41 (0.39-0.43) | <0.001 | 0.45 (0.43-0.48) | <0.001 |
| Per 10 score increase | 274608 | 8915/3564477 | 0.74 (0.73-0.75) | <0.001 | 0.76 (0.75-0.78) | <0.001 |

HR, hazard ratio; CI, confidence interval

Model 1: Adjusted for age and sex;

Model 2: Further adjusted for assessment center, years of education, income levels, employment status, Index of Multiple Deprivation, alcohol consumption, and health behavior score


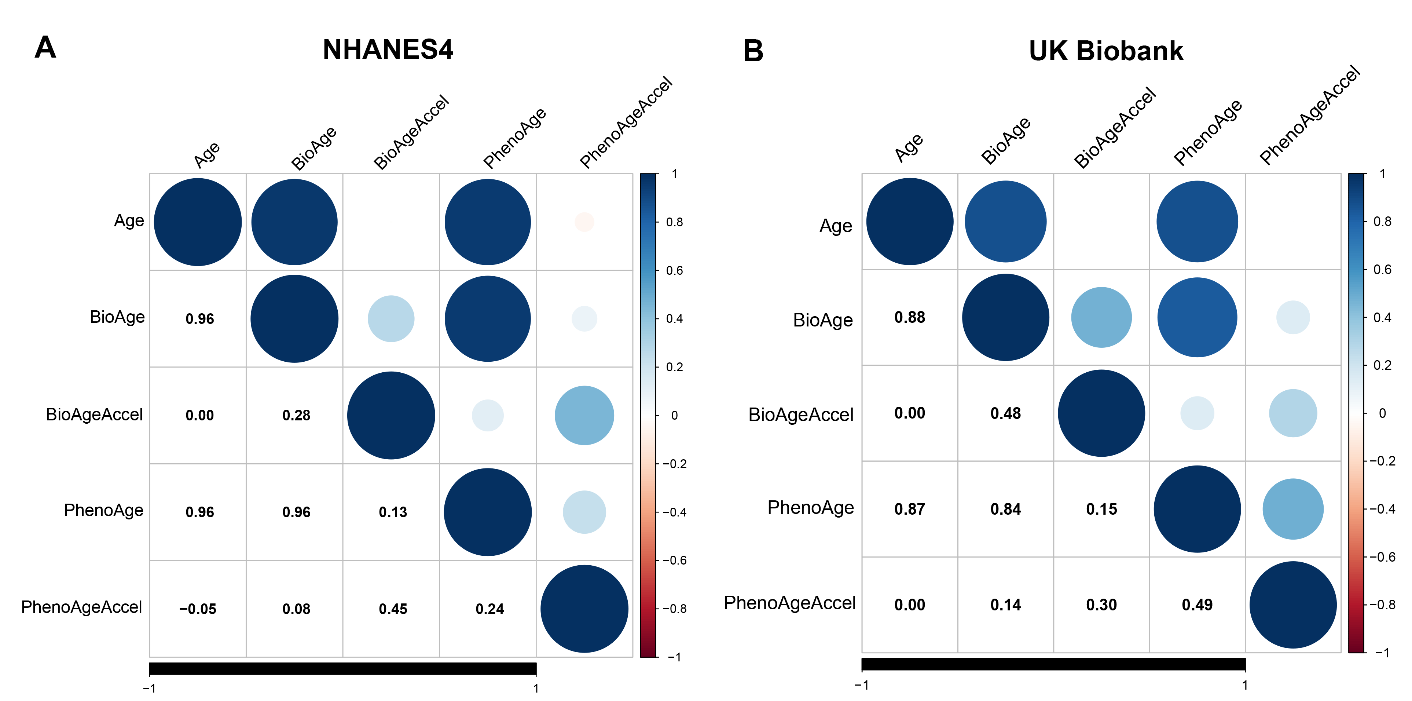


**Figure S1. Correlations between biological age measures and chronological age in UK Biobank.**

BioAgeAccel, Biological Age acceleration; PhenoAgeAccel, Phenotypic Age acceleration


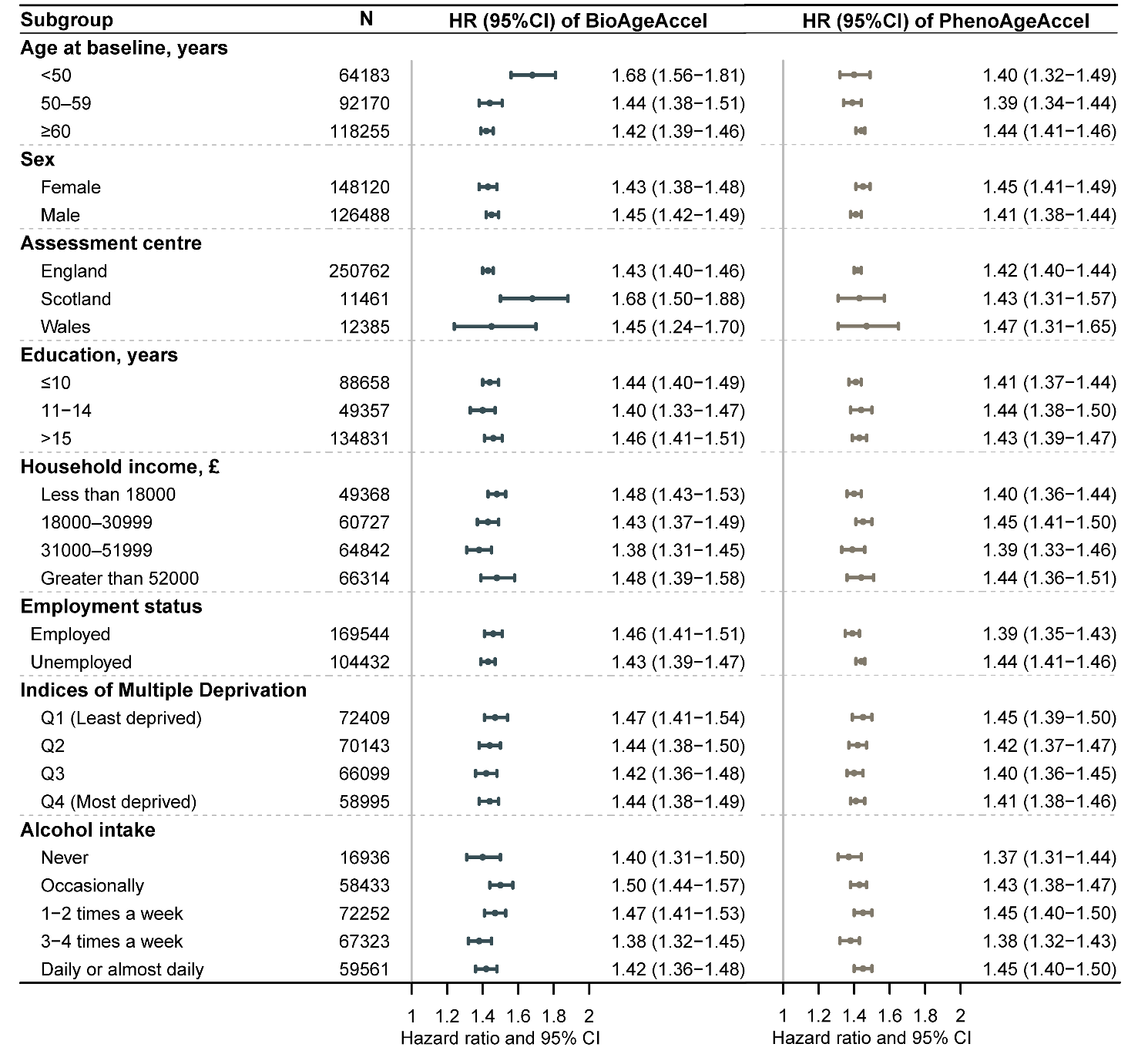


**Figure S2. Association of biological age accelerations and the risk of incident heart failure stratified by potential risk factors.**

BioAgeAccel, Biological Age acceleration; PhenoAgeAccel, Phenotypic Age acceleration; HR, hazard ratio; CI, confidence interval.


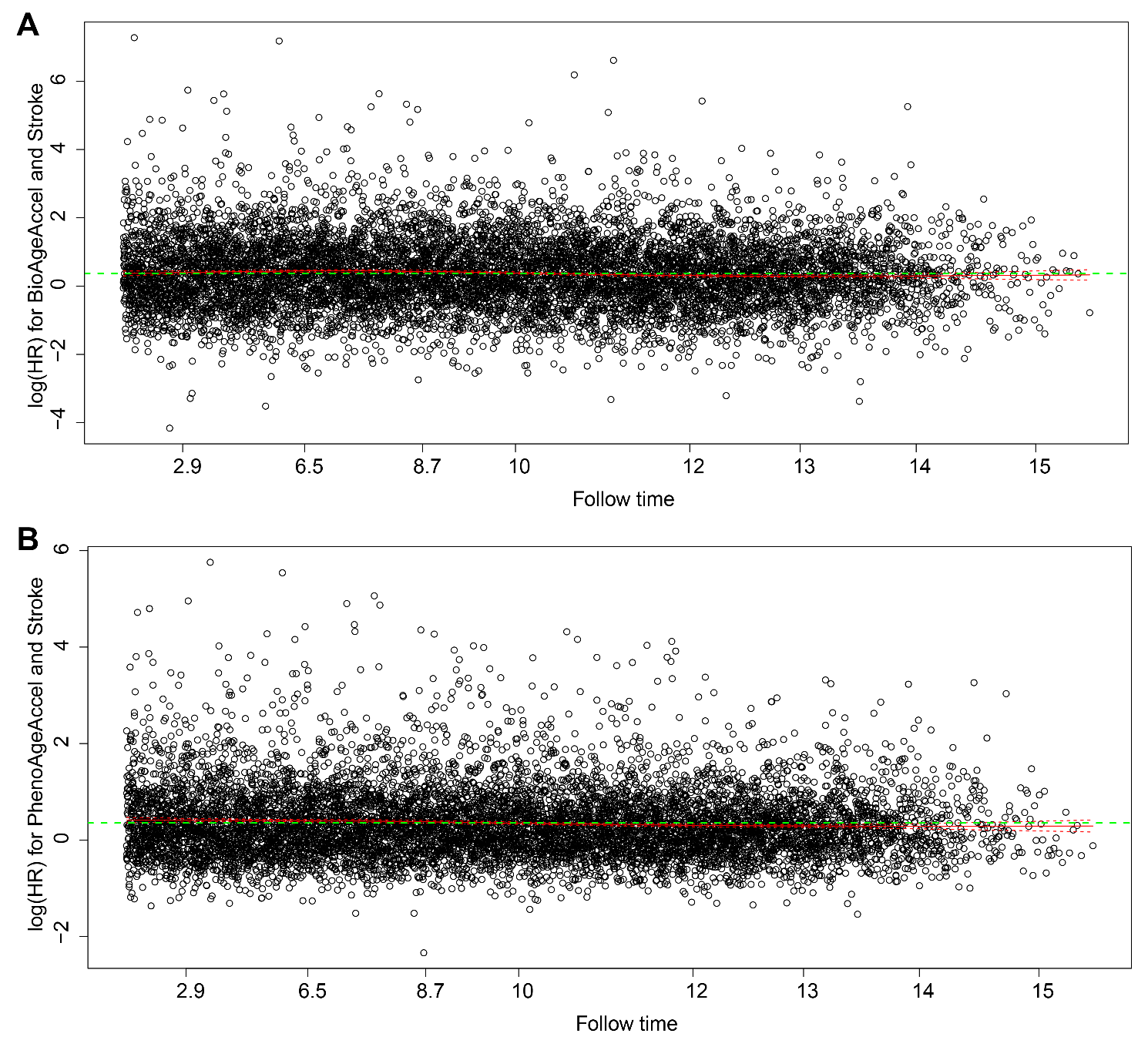


**Figure S3. Scaling Schoenfeld residuals and time-based logarithmic HR plots of biological age accelerations.**

BioAgeAccel, Biological Age acceleration; PhenoAgeAccel, Phenotypic Age acceleration; HR, hazard ratio


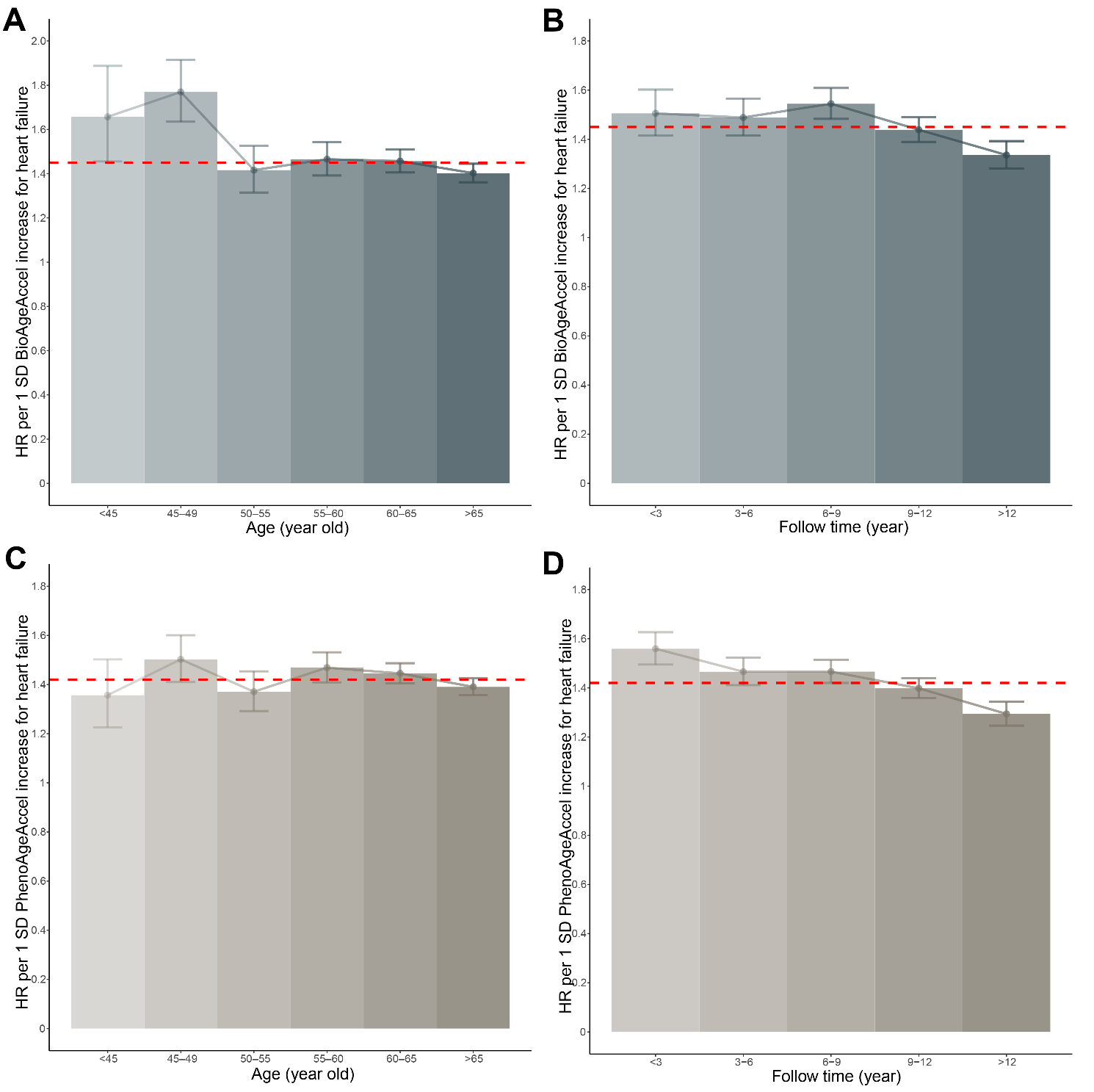


**Figure S4. Association between biological age accelerations and the risk of incident heart failure using time-varying model with interaction terms between BioAgeAccel or PhenoAgeAccel and age (in 5-year intervals) (A and C) or between BioAgeAccel or PhenoAgeAccel and follow-up time (in 3-year intervals) (B and D).**

BioAgeAccel, Biological Age acceleration; PhenoAgeAccel, Phenotypic Age acceleration; HR, hazard ratio; CI, confidence interval; SD, standard deviations


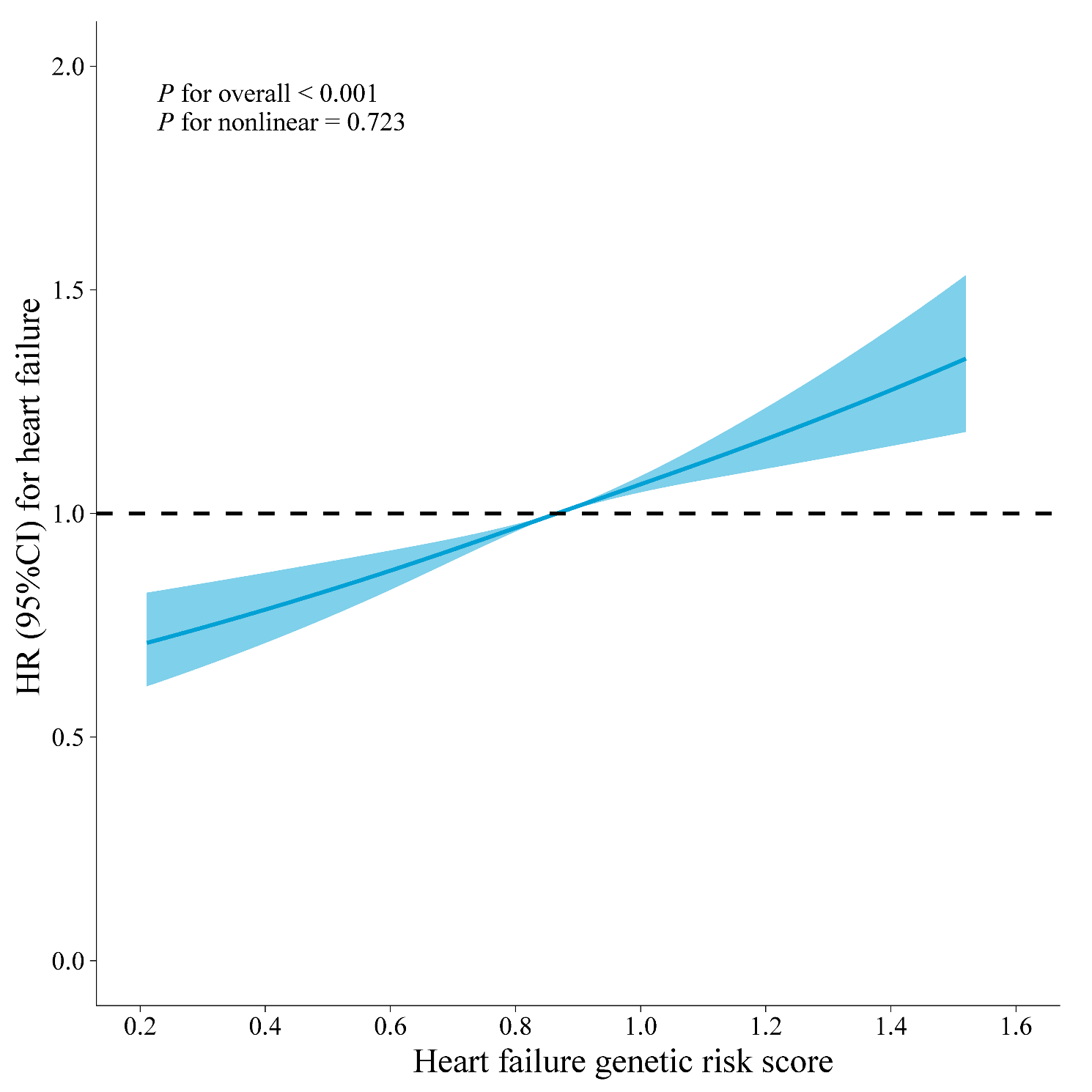


**Figure S5. Association between genetic risk scores and the risk of incident heart failure using restricted cubic splines models with three knots.**

HR, hazard ratio; CI, confidence interval

Adjusted for age, sex, assessment center, years of education, income levels, employment status, Index of Multiple Deprivation, alcohol consumption, health behavior score, genotyping array, and the first ten genetic principal components


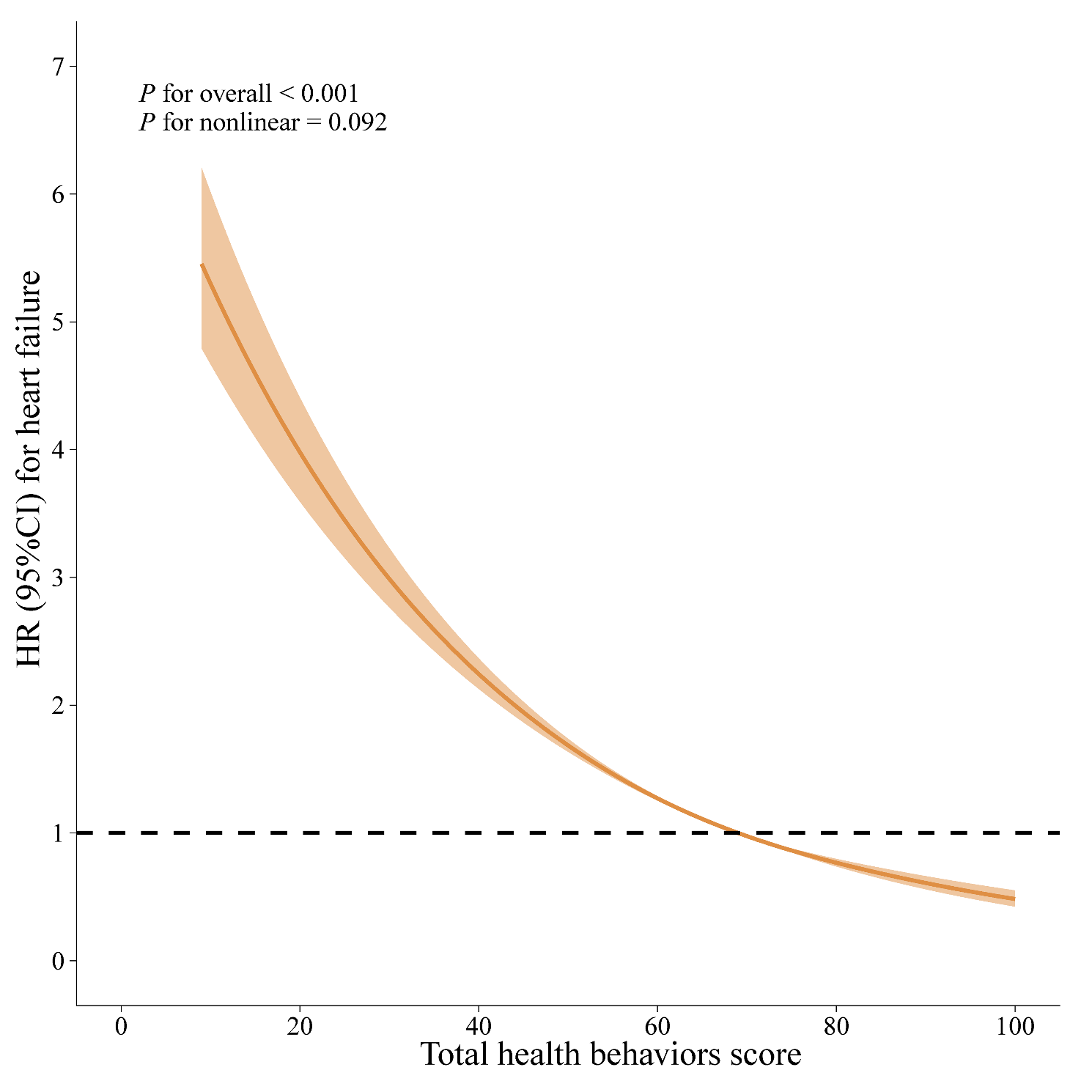


**Figure S6. Association between total health behavior scores and the risk of incident heart failure using restricted cubic splines models with three knots.**

HR, hazard ratio; CI, confidence interval

Adjusted for age, sex, assessment center, years of education, income levels, employment status, Index of Multiple Deprivation, and alcohol consumption
